# Esophageal pressure as estimation of pleural pressure: a study in a model of eviscerated chest

**DOI:** 10.1186/s12871-024-02806-0

**Published:** 2024-11-14

**Authors:** Gaetano Florio, Eleonora Carlesso, Francesco Mojoli, Fabiana Madotto, Luigi Vivona, Chiara Minaudo, Michele Battistin, Sebastiano Maria Colombo, Stefano Gatti, Simone Sosio, Antonio Pesenti, Giacomo Grasselli, Alberto Zanella

**Affiliations:** 1 Dipartimento Area Emergenza Urgenza, Fondazione IRCCS Ca’ Granda Ospedale Maggiore Policlinico, Milan, Italy; 2 Department of Pathophysiology and Transplantation, University of Milan, Via Francesco Sforza 35, Milan, 20122 Italy; 3 Anesthesia and Intensive Care, Fondazione IRCCS Policlinico S. Matteo, Pavia, Italy; 4 Dipartimento Scienze Clinico-Chirurgiche, Diagnostiche E Pediatriche, University of Pavia, Pavia, Italy; 5 Department of Anaesthesia and Intensive Care, San Giuseppe Hospital, IRCCS MultiMedica, Milan, Italy; 6 Center for Preclinical Research, Fondazione IRCCS Ca’ Granda – Ospedale Maggiore Policlinico, Milan, Italy; 7 Anesthesia and Intensive Care Unit, Fondazione IRCCS San Gerardo Dei Tintori, Monza, Italy

**Keywords:** Esophageal pressure, Intrathoracic pressure, Mechanical ventilation, Correction, Esophageal balloon, Transpulmonary pressure

## Abstract

**Background:**

Transpulmonary pressure is the effective pressure across the lung parenchyma and has been proposed as a guide for mechanical ventilation. The pleural pressure is challenging to directly measure in clinical setting and esophageal manometry using esophageal balloon catheters was suggested for estimation. However, the accuracy of using esophageal pressure to estimate pleural pressure is debated due to variability in the mechanical properties of respiratory system, esophagus and esophageal catheter. Furthermore, while a vertical pleural pressure gradient exists across lung regions, esophageal pressure balloon provides a single value, representing, at most, the pressure surrounding the esophagus.

**Methods:**

In a swine model with a preserved esophagus and a single homogenous, easily measurable intrathoracic pressure, we evaluated esophageal pressure’s agreement with intrathoracic pressure at different positive end-expiratory pressure (PEEP) levels (0, 5, 10, 15 cmH_2_O). We assessed the improvement of measurement accuracy by correcting absolute esophageal values using a previously described technique, that accounts for the pressure generated by the esophageal wall in response to esophageal balloon inflation.

The study involved five swine, wherein two different esophageal catheters were used alongside the four distinct PEEP levels. Swings, uncorrected and corrected absolute esophageal pressures (end-inspiratory, end-expiratory) were compared with their respective intrathoracic pressures. The effect of correction technique was assessed with manual incremental step inflation procedure.

**Results:**

We found that both catheters significantly overestimated absolute esophageal pressure compared to intrathoracic pressure (5.01 ± 3.32 and 6.06 ± 5.62 cmH_2_O at end-expiration and end-inspiration, respectively), with error increasing at higher positive end-expiratory pressure levels (end-expiration: 2.36 ± 2.03, 3.77 ± 1.37, 6.24 ± 2.51 and 7.69 ± 4.02 for each PEEP level, *P* < 0.0001; end-inspiration: 1.71 ± 2.10, 3.70 ± 1.73, 7.67 ± 3.62 and 11.14 ± 7.60 for each PEEP level, *P* = 0.0004).

Applying the correction technique significantly improved agreement for absolute values (0.82 ± 1.62 and 1.86 ± 3.94 cmH_2_O at end-expiration and end-inspiration, respectively). Esophageal pressure swings accurately estimated intrathoracic pressure swings at low-medium intrathoracic pressures (-0.64 ± 0.62, -0.07 ± 0.53, 1.43 ± 1.51, and 3.45 ± 3.94 at PEEP 0, 5, 10 and 15 cmH_2_O, respectively; *P* = 0.0197).

**Conclusions:**

The correction technique, based on the mechanical response of esophageal wall to the balloon inflation, is fundamental for obtaining reliable estimations of absolute intrathoracic pressure values, and for ensuring its correct application in clinical setting.

**Supplementary Information:**

The online version contains supplementary material available at 10.1186/s12871-024-02806-0.

## Background

Mechanical ventilation represents the cornerstone therapy for acute respiratory failure and multiple research efforts aimed to decrease its harm and to improve patient survival [1]. It has been demonstrated that tidal volume < 6 mL/kg, plateau pressure < 30 cmH_2_O, driving pressure < 14 cmH_2_O and mechanical power < 17 J/min are associated with decreased mortality [2–5]. However, transpulmonary pressure (the difference between alveolar and pleural/intrathoracic pressures) describes the stress directly acting on the lung parenchyma and could guide more accurately protective ventilation [6]. According to some reports, mechanical ventilation should be set to obtain adequate oxygenation minimizing alveolar collapse and overdistension [7–10]. Positive end-expiratory pressure (PEEP) should be set to keep a slightly positive end-expiratory transpulmonary pressure while end-inspiratory transpulmonary pressure should be used as safety limit [7–10].

Due to the challenge of direct measurement of intrathoracic pressure, esophageal pressure has been proposed as surrogate [6]. The technique uses an air-filled balloon placed in the mid-lower part of the esophagus and assumes that the pressure surrounding the esophagus is transmitted to the balloon. However, intrathoracic pressure is not homogeneously distributed in the chest and the esophageal pressure better represents the intrathoracic pressure in the regions adjacent the esophagus [11–15]. Furthermore, the mechanical properties of the respiratory system, esophageal wall, and catheter affect the measurement [16–19]. Moreover, the balloon inflation volume could lead to insufficient (low filling volumes) or excessive (high filling volumes) transmission of absolute intrathoracic pressure values [17–20]. It has been proposed to determine the optimal catheter filling volume (V_BEST_) by progressively inflating the balloon and selecting the volume associated with the maximal inspiratory to expiratory pressure difference (swing) within the quasi-linear range of the volume-pressure (VP) curve.

Some authors do not consider reliable the measurement of absolute values and proposed to use only esophageal pressure swings [7]. To make the estimation of absolute values more reliable, multiple correction strategies have been developed, mainly based on the progressive inflation of the balloon and on the construction of VP curves [17, 21, 22]. Mojoli et al. tested a correction technique based on V_BEST_ and on the subtraction of the artifactual pressure generated by the esophagus wall due to the balloon inflation [22]. To investigate the reliability of esophageal pressure as estimation of intrathoracic pressure, we developed a swine model of eviscerated chest, preserving the esophagus. Specifically, the model was developed to reflect, as much as possible, chest wall and esophageal physiology and mechanics and to obtain a single, homogeneous, easy measurable value of intrathoracic pressure surrounding the esophagus and to compare it with the correspondent esophageal pressure. The aims of the study are: 1) to evaluate the agreement of esophageal pressure with intrathoracic pressure at different PEEP levels; 2) to assess the improvement of the measurement filling the esophageal balloon at an optimal volume and subtracting the artifactual pressure generated by the esophagus wall.

## Methods

This ex vivo study was conducted after a 24-h study evaluating a low-flow extracorporeal carbon dioxide removal (ECCO_2_R) technique on five swine following their euthanasia. As per Italian law, no dedicated approval was required for this ex vivo study. The preceding in vivo ECCO_2_R study, which adhered to ministerial guidelines, was approved by the Italian Ministry of Health (permit number: 463/2018-PR, June 22, 2018). Animals received humane care in compliance with European Union Directive 2010/63/EU on the protection of animals used for scientific studies and Italian Legislative Decree 26/2014.

Five pigs (44 ± 5 kg), purchased from an Agricultural Company by Fondazione IRCCS Ca’ Granda Ospedale Maggiore Policlinico of Milan were studied. In compliance with local recommendations, pigs arrived at the experimental facility the day before the start of the study and fasted overnight with free access to water. Anesthesia was maintained during the entire in vivo study and during euthanasia by continuous intravascular infusion of propofol (4–12 mg/kg/h), medetomidine (0.2 mg/h) and pancuronium (12 mg/h) for muscular paralysis. At the end of the in vivo experiment, animals were euthanized by intravenous injection of potassium chloride 40 mEq.

**Fig. 1:**
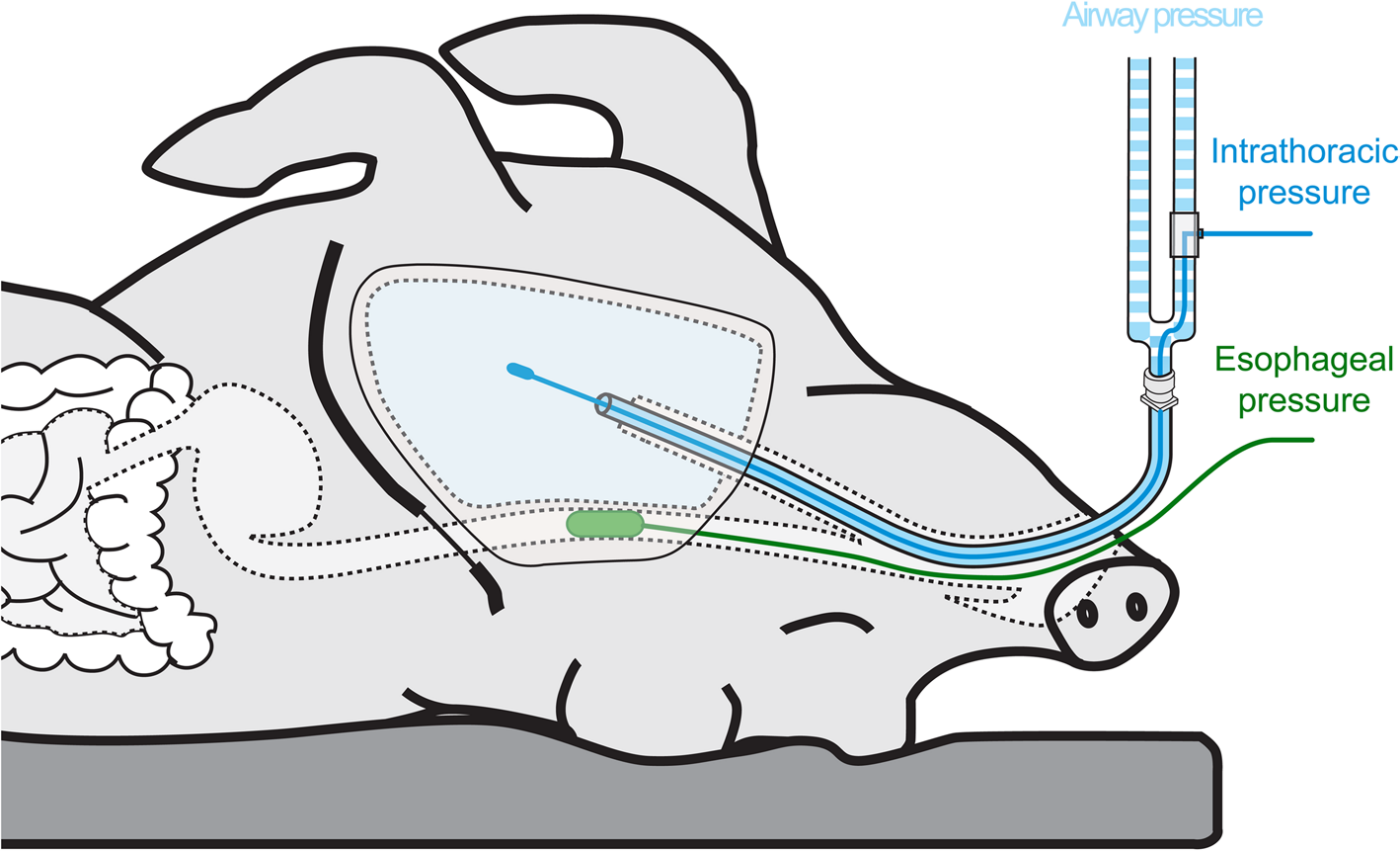
Swine model of eviscerated chest. Figure shows the swine model of eviscerated chest where all the thoracic organs were removed while preserving the esophagus. The light blue area represents the inelastic plastic bag adhering to the chest wall and sealed to an endotracheal tube. The intrathoracic pressure was measured through a dedicated catheter (in blue). The esophageal pressure was measured through two different esophageal catheters (in green)

After sternotomy, the thoracic organs were removed while preserving the esophagus. An inelastic plastic bag with a resting volume significantly greater than the intrathoracic volume was tightly sealed to an endotracheal tube and introduced in the thorax adhering to the chest wall. The endotracheal tube was connected to the ventilator (GE, Datex Ohmeda Engström Carestation, General Electric, Madison, WI, USA). The ventilator settings were: volume-controlled mode, tidal volume 10 mL/kg, respiratory rate 10/min, I:E 1:3. PEEP was tested at 0, 5, 10 and 15 cmH_2_O. Intrathoracic pressure was measured through a small rigid catheter placed inside the bag through endotracheal tube (Fig. 1). Two esophageal catheters were tested: Cooper (Cooper Surgical, Trumbull, CT, USA) and Nutrivent (Sidam Srl, Modena, Italy). Esophageal balloon was placed under direct inspection while the chest was open and then assessed with Baydur test [21]. VP curves of the balloon catheters were obtained during manual incremental step inflation (10 progressive inflation steps up to maximum volume [2.8 mL and 10 mL for Cooper and Nutrivent, respectively]). PEEP and catheters were randomized within the same swine (Additional file 1, Fig. E1). To assess changes in esophagus + esophageal balloon elastance (E_ES_), possibly due to esophagus deterioration throughout the study, VP curves without ventilation were obtained at the beginning and at the end of the study. Curves were recorded with LabChart (ADInstruments Inc, Colorado Springs, CO, USA). Esophageal pressure values at end-inspiration, end-expiration and swings were extracted from an average breath obtained from 5 breaths at each step of the VP curve.

### Data definition and statistical analysis

We consider V_BEST_ as the esophageal balloon filling volume, within the quasi-linear range of the end-expiratory VP curve, maximizing esophageal pressure swing (inspiratory-to-expiratory) [22]. The quasi-linear range, delimited by V_MIN_ and V_MAX_, was obtained through a sigmoidal model [23, 24] (Additional file 1, Fig. E2). The filling volume recommended by the catheter’s producer (V_producer_) was 4 mL and 1 mL for Nutrivent and Cooper, respectively.

Generalized linear mixed models with fixed and random effects were used to test the effect of PEEP or catheter on V_BEST_, V_MIN_, chest wall compliance, E_ES_ and to compare intrathoracic and esophageal pressure measured at V_producer_ at different PEEP levels. Uncorrected esophageal pressures were measured at V_BEST_ and at V_producer_ and then corrected to consider the effect of esophagus wall [22]. Specifically, the end-expiratory and end-inspiratory esophageal pressures obtained at V_BEST_ were corrected subtracting the artifactual increase of pressure generated by the esophagus wall (P_EW_) due to the balloon filling and calculated as:

$${{\mathrm P}_{\mathrm{EW}}=\left({\mathrm V}_{\mathrm x}\;-\;{\mathrm V}_{\mathrm{MIN}}\right)\;\times\;{\mathrm E}_{\mathrm{ES}}}$$

where Vx was any filling volume above V_MIN_ and E_ES_, the elastance of the system esophagus + esophageal balloon, was computed as the ratio of (P_MAX_ – P_MIN_) / (V_MAX_ – V_MIN_), the slope of the quasi-linear section of the expiratory curve. The same correction procedure was also applied to end-expiratory and end-inspiratory esophageal pressures obtained at the balloon filling volume suggested by the producer. As previously described, the same P_EW_ correction factor was applied to correct both the end-expiratory and end-inspiratory esophageal pressure values.

Data were reported as mean ± standard deviation (SD). The relationships between intrathoracic and esophageal pressures were modeled by univariate linear regressions according to catheter, correction, and PEEP. The effects of catheter, PEEP, correction, and interactions on the esophageal and intrathoracic pressure difference were evaluated by generalized linear mixed models with fixed and random effects (including intercept). Pairwise comparisons were tested with Bonferroni’s correction.

Wilcoxon Signed Rank Test was used to compare elastance values observed at the beginning and the end of the study. See Additional file 1 for details.

All statistical tests were two sided (α = 0.05). Analyses were performed using SAS software v.9.4 (SAS Institute, NC, USA) and SigmaPlot (Systat Software, San Jose, CA).

## Results

All measurements were collected in all the five swine.

**Fig. 2:**
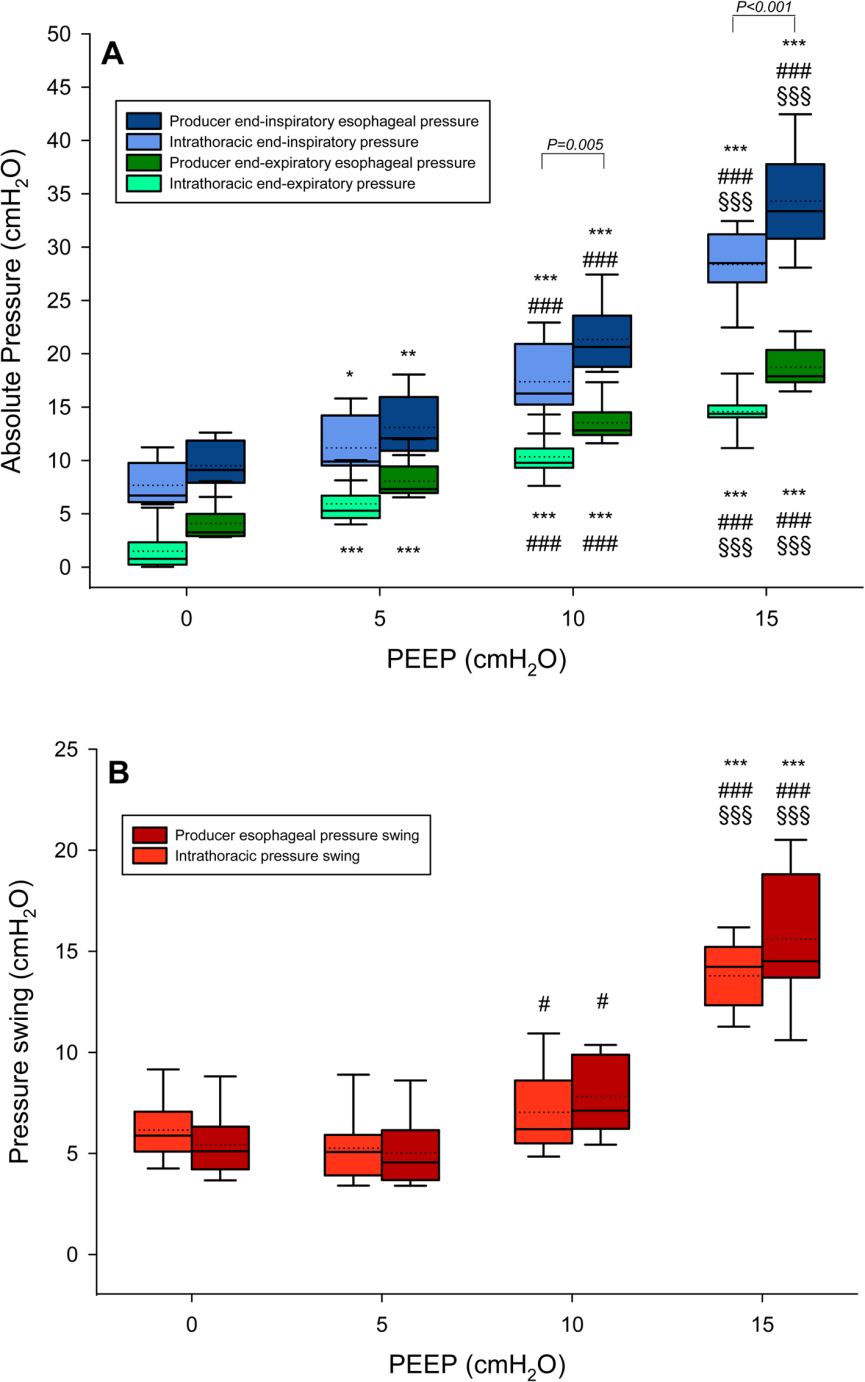
Distributions of esophageal (esophageal balloon inflated at V_producer_) and intrathoracic pressure at different PEEP levels. Boxplot representation at different PEEP levels of absolute esophageal and intrathoracic pressures (panel **A**) and pressure swings (panel **B**) measured when the esophageal balloon was inflated at the volume suggested by the producer (4 mL for Nutrivent, 1 mL for Cooper) during the VP curves in the study population (values from both catheters together were included). Boxplots represent median, 10th, 25th, 75th and 90th percentiles as boxes with error bars; dotted lines represent the mean values. Panel **A**: blue boxes represent end-inspiratory esophageal pressure at the volume suggested by the producer; light-blue boxes represent intrathoracic end-inspiratory pressure; dark green boxes represent end-expiratory esophageal pressure at the volume suggested by the producer; green boxes represent intrathoracic end-expiratory pressure. Panel **B**: dark red boxes represent esophageal pressure swings at the volume suggested by the producer; red boxes represent intrathoracic pressure swings. * *P* < 0.05 vs PEEP 0 cmH_2_O; ** *P* < 0.01 vs PEEP 0 cmH_2_O; *** *P* < 0.001 vs PEEP 0 cmH_2_O; # *P* < 0.05 vs PEEP 5 cmH_2_O; ### *P* < 0.001 vs PEEP 5 cmH_2_O; §§§ *P* < 0.0001 vs PEEP 10 cmH_2_O

The pigs were ventilated with a tidal volume of 452 ± 50 mL. Both end-expiratory and end-inspiratory intrathoracic and esophageal pressures (measured when the balloon was inflated at V_producer_) increased with the PEEP level (*P* < 0.0001 for both). Moreover, mean end-expiratory intrathoracic pressures were 1.5 ± 1.9, 5.9 ± 2.0, 10.3 ± 2.0 and 14.6 ± 1.9 cmH_2_O at PEEP of 0, 5, 10 and 15 cmH_2_O, respectively, while end expiratory esophageal pressures resulted higher than the corresponding intrathoracic pressures (*P* = 0.01) and were 4.1 ± 1.7, 8.1 ± 1.8, 13.5 ± 1.8, 18.7 ± 1.9 cmH_2_O (Fig. 2, Panel A). Similarly, end-inspiratory esophageal pressure was higher than intrathoracic pressure at PEEP 10 (*P* = 0.005) and 15 cmH_2_O (*P* < 0.0001) (Fig. 2, Panel A). Intrathoracic and esophageal pressure swings at the same level of PEEP were not different (*P* = 0.42) but their values increased at higher PEEP (*P* < 0.0001) (Fig. 2, Panel B). The chest wall compliance was 77.8 ± 22.8, 94.2 ± 29.7, 69.5 ± 20.3, 33.4 ± 6.2 mL/cmH_2_O at PEEP 0, 5, 10, and 15 cmH_2_O, respectively (*P* < 0.0001, Fig. E3). See Additional file 1 for further results.

Average end-expiratory and end-inspiratory volume-pressure curves of the balloon for each catheter and PEEP level are reported in the Additional file 1 (Fig. E4-E5). Figures E6-E15 show the curve fittings the experimental data points, with the indication of the quasi-linear section of the end-expiratory curve and the selected V_BEST_.

V_BEST_ for the Cooper catheter was 1.3 ± 0.8, 2.1 ± 0.1, 2.1 ± 0.0 and 2.2 ± 0.1 mL at PEEP 0, 5, 10 and 15 cmH_2_O, respectively (*P* = 0.017; *P* < 0.05, PEEP 15 vs PEEP 0), while for the Nutrivent catheter V_BEST_ was 3.2 ± 2.3, 5.1 ± 1.8, 6.0 ± 0.1, 6.0 ± 0.1 mL at PEEP 0, 5, 10 and 15 cmH_2_O, respectively (*P* = 0.018; *P* < 0.05, PEEP 15 and PEEP 10 vs PEEP 0). V_MIN_ for the Cooper catheter was 0.4 ± 0.2, 0.5 ± 0.1, 0.5 ± 0.0 and 0.6 ± 0.0 mL at PEEP 0, 5, 10 and 15 cmH_2_O, respectively (*P* = 0.056), while for the Nutrivent catheter V_MIN_ was 1.4 ± 0.2, 1.4 ± 0.2, 1.3 ± 0.3, 1.4 ± 0.1 mL at PEEP 0, 5, 10 and 15 cmH_2_O, respectively (*P* = 0.734). See Additional file 1 (Tables E1-E6) for further data.

The mean esophagus + esophageal balloon elastance (computed on the expiratory VP curve) was 2.5 ± 0.85 and 1.30 ± 0.58 cmH_2_O/mL with Cooper and Nutrivent catheter, respectively (*P* = 0.043). For the Cooper catheter esophagus + esophageal balloon elastance was 2.2 ± 1.0, 2.2 ± 0.4, 2.7 ± 0.8 and 2.9 ± 1.0 cmH_2_O/mL at PEEP 0, 5, 10, and 15 cmH_2_O respectively (*P* = 0.031). For the Nutrivent catheter esophagus + esophageal balloon elastance was 1.1 ± 0.4, 1.0 ± 0.4, 1.4 ± 0.7 and 1.7 ± 0.7 cmH_2_O/mL at PEEP 0, 5, 10, and 15 cmH_2_O respectively (*P* = 0.013; *P* < 0.05, PEEP 0 and PEEP 5 vs PEEP 15).

### Corrected measurement of end-expiratory esophageal pressure has increased accuracy in estimating end-expiratory intrathoracic pressure

**Fig. 3:**
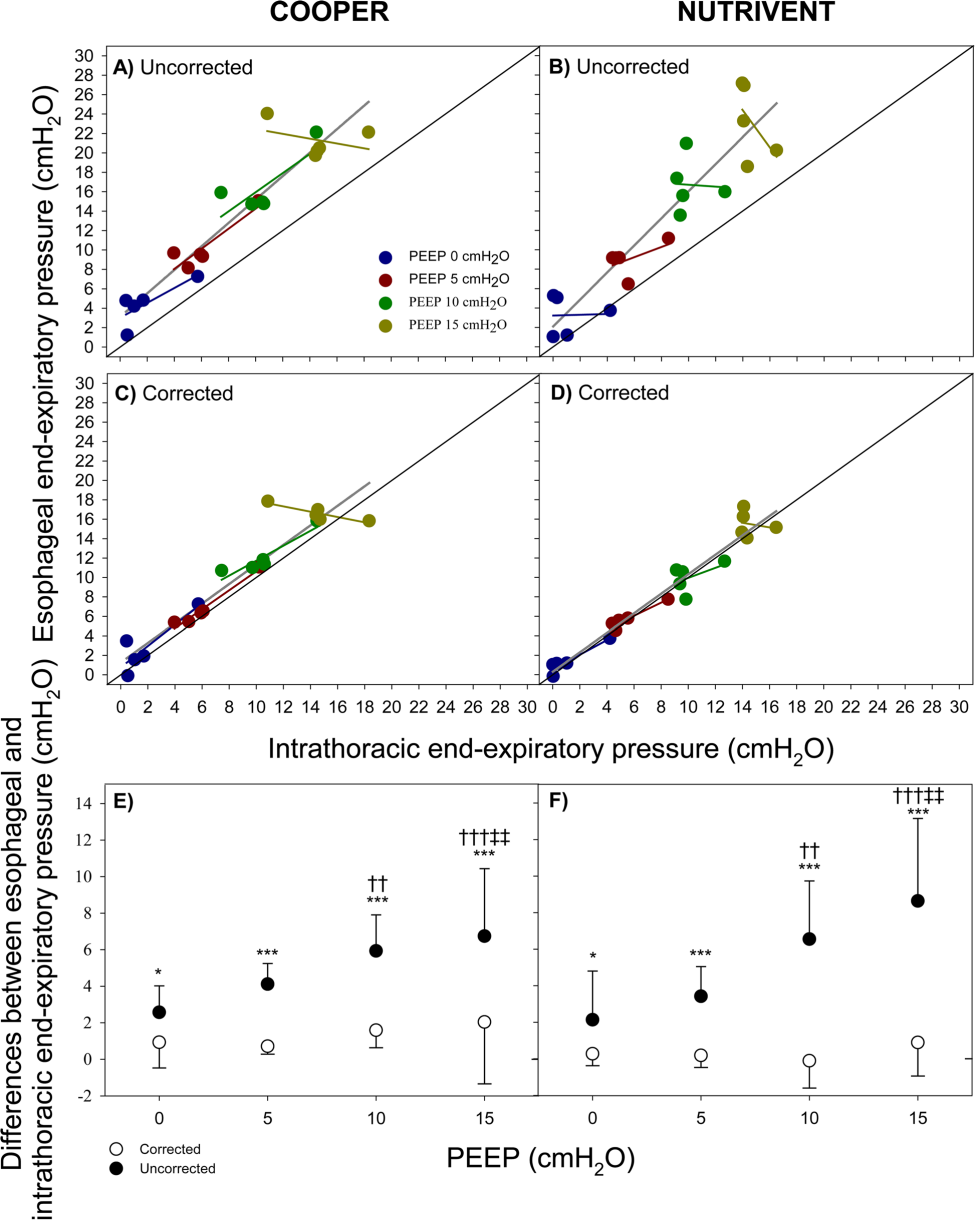
Relationship between end-expiratory esophageal pressure and end-expiratory intrathoracic pressure (esophageal balloon inflated at V_BEST_). Figure shows the relationship (overall and according to different PEEP levels) between end-expiratory esophageal pressure and end-expiratory intrathoracic pressure (panels **A**, **B**, **C**, **D**) and their difference (panels **E** and **F**, white dots represent corrected values while black dots represent uncorrected values) according to the tested catheters (Cooper, panels **A**, **C**, **E**; Nutrivent panels **B**, **D**, **F**). Panels **A** and **B** refer to uncorrected end-expiratory esophageal pressure while panels **C** and **D** refer to corrected values. Blue color represents PEEP 0 cmH_2_O; dark-red color represents PEEP 5 cmH_2_O; green color represents PEEP 10 cmH_2_O; dark-yellow color represents PEEP 15 cmH_2_O. Blue, dark-red, green and dark-yellow lines represent linear regressions at different PEEP values, dark-grey line represents linear regression at all PEEP levels. Black continuous line represents the identity line. * *P* < 0.05 vs “corrected”; *** *P* < 0.001 vs “corrected”; †† *P* < 0.01 vs PEEP 0; ††† *P* < 0.001 vs PEEP 0; ‡‡ *P* < 0.01 vs PEEP 5

Figure 3 shows the relationship (overall and at each level of PEEP) between end-expiratory pressure at V_BEST_ and intrathoracic pressure with the two tested catheters. Panels A and B report the uncorrected values while Panels C and D represent the corrected values. Both uncorrected and corrected esophageal pressure values correlate with intrathoracic pressure values but corrected esophageal pressure better approximate intrathoracic pressure. Indeed, the overall difference between esophageal pressure and intrathoracic pressure was 5.01 ± 3.32 and 0.82 ± 1.62 cmH_2_O (*P* = 0.0003) for uncorrected and corrected pressures, respectively (Panels E and F).

The difference between uncorrected esophageal pressure and intrathoracic pressures increased with PEEP (2.36 ± 2.03, 3.77 ± 1.37, 6.24 ± 2.51 and 7.69 ± 4.02 at PEEP 0, 5, 10 and 15 cmH_2_O, respectively; *P* < 0.0001) while the differences computed with corrected esophageal pressure did not (0.60 ± 1.09, 0.45 ± 0.60, 0.74 ± 1.48 and 1.47 ± 2.64 at PEEP 0, 5, 10 and 15 cmH_2_O, respectively; *P* = 0.8115). No effect of catheter was detected in the mean difference estimation (*P* = 0.4608).

### Corrected measurement of end-inspiratory esophageal pressure has increased accuracy in estimating end-inspiratory intrathoracic pressure

**Fig. 4:**
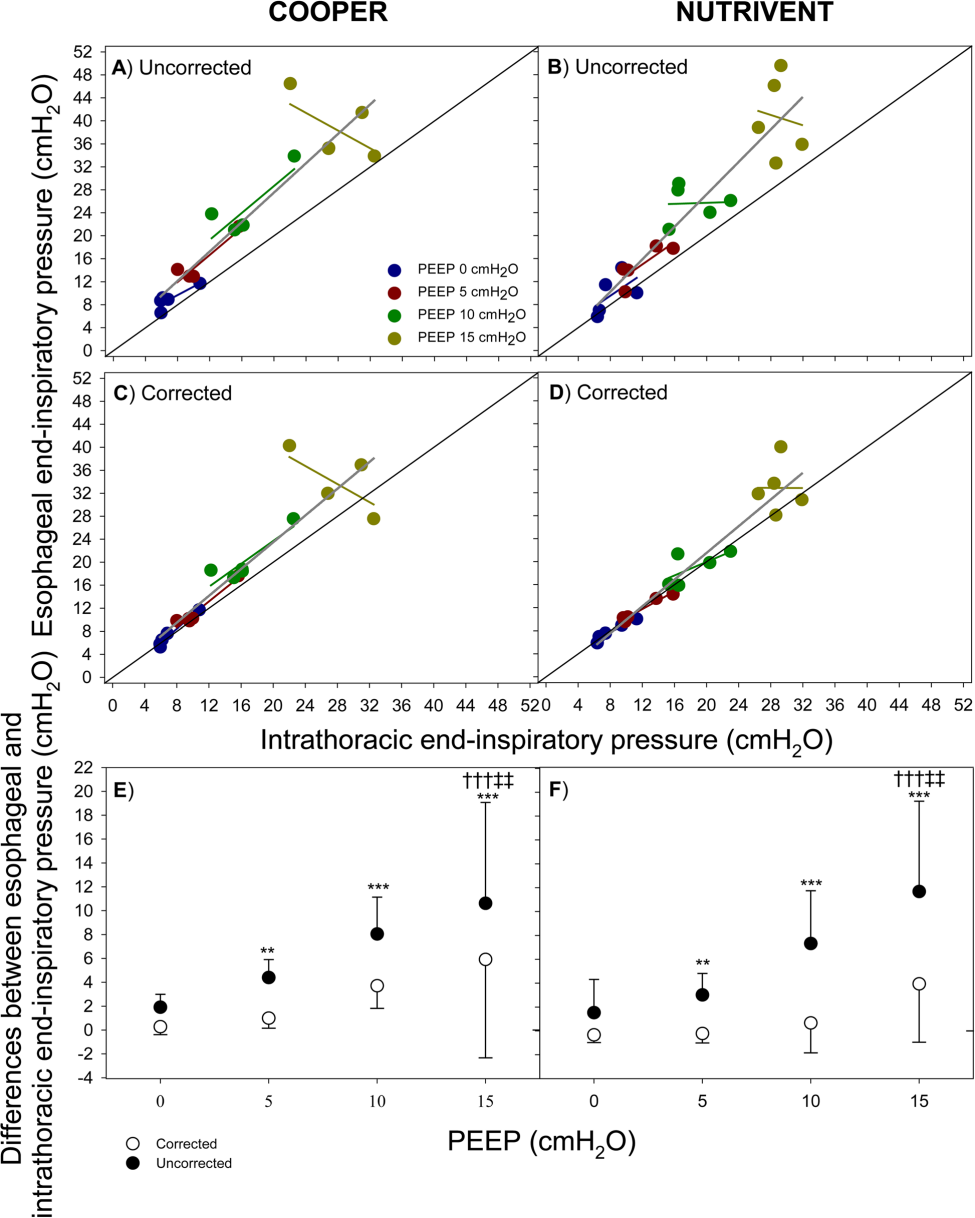
Relationship between end-inspiratory esophageal pressure and end-inspiratory intrathoracic pressure (esophageal balloon inflated at V_BEST_). Figure shows the relationship (overall and according to different PEEP levels) between end-inspiratory esophageal pressure and end-inspiratory intrathoracic pressure (panels **A**, **B**, **C**, **D**) and their difference (panels **E** and **F** white dots represent corrected values while black dots represent uncorrected values) according to the tested catheters (Cooper, panels **A**, **C**, **E**; Nutrivent panels **B**, **D**, **F**). Panels **A** and **B** refer to end- inspiratory esophageal pressure while panels **C** and **D** refer to corrected values. Blue color represents PEEP 0 cmH_2_O; dark-red color represents PEEP 5 cmH_2_O; green color represents PEEP 10 cmH_2_O; dark-yellow color represents PEEP 15 cmH_2_O. Blue, dark-red, green and dark-yellow lines represent linear regressions at different PEEP values, dark-grey line represents linear regression at all PEEP levels. Black continuous line represents the identity line. ** *P* < 0.01 vs “corrected”; *** *P* < 0.001 vs “corrected”; ††† *P* < 0.001 vs PEEP 0; ‡‡ *P* < 0.01 vs PEEP 5

Figure 4 shows the relationship (overall and at each level of PEEP) between end-inspiratory pressure at V_BEST_ and intrathoracic pressure with the two tested catheters. Panels A and B report the uncorrected values while Panels C and D represent the corrected values. Similarly, to end-expiratory values, both uncorrected and corrected esophageal pressure values correlate with intrathoracic pressure values but corrected esophageal pressure better approximate intrathoracic pressure. Indeed, the overall difference between esophageal pressure and intrathoracic pressure was 6.06 ± 5.62 to 1.86 ± 3.94 cmH_2_O (*P* = 0.0010) for uncorrected and corrected pressures, respectively (Panels E and F).

The differences computed with uncorrected esophageal pressure significantly increased with PEEP (1.71 ± 2.10, 3.70 ± 1.73, 7.67 ± 3.62 and 11.14 ± 7.60 at PEEP 0, 5, 10 and 15 cmH_2_O, respectively; *P* = 0.0004) while the differences computed with corrected esophageal pressure did not (-0.04 ± 0.69, 0.38 ± 1.00, 2.17 ± 2.63 and 4.92 ± 6.47 at PEEP 0, 5, 10 and 15 cmH_2_O, respectively; *P* = 0.1155). No effect of catheter was detected on the mean difference estimation (*P* = 0.1651).

### Esophageal pressure swings accurately estimate intrathoracic pressure swings

**Fig. 5:**
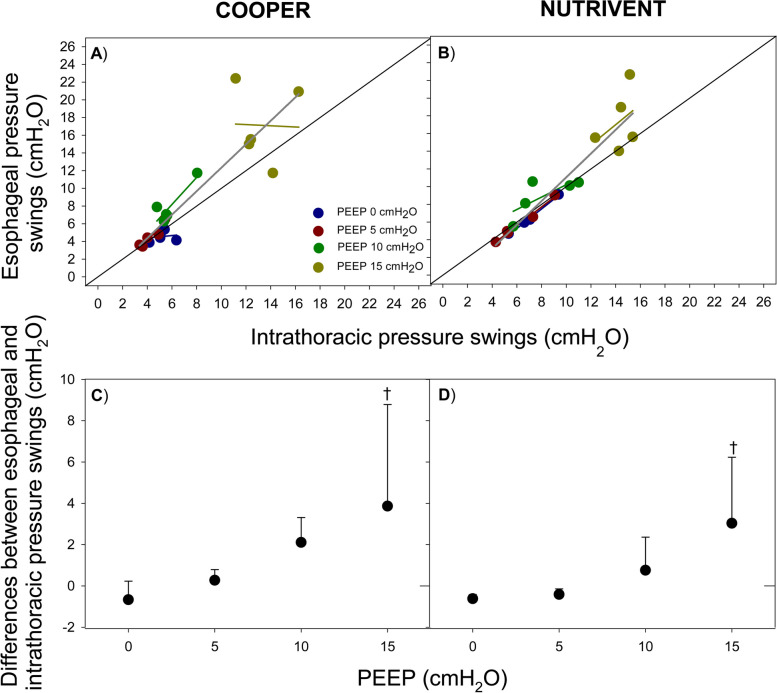
Relationship between esophageal pressure swings and intrathoracic pressure swings (esophageal balloon inflated at V_BEST_). Figure shows the relationship (overall and according to different PEEP levels) between esophageal pressure swings and intrathoracic pressure swings (panels **A**, **B**) and their difference (panels **C** and **D**) according to the tested catheters (Cooper, panels **A**, **C**; Nutrivent panels **B**, **D**). Blue color represents PEEP 0 cmH_2_O; dark-red color represents PEEP 5 cmH_2_O; green color represents PEEP 10 cmH_2_O; dark-yellow color represents PEEP 15 cmH_2_O. Blue, dark-red, green and dark-yellow lines represent linear regressions at different PEEP values, dark-grey line represents linear regression at all PEEP levels. Black continuous line represents the identity line. † *P* < 0.05 vs PEEP 0

Figure 5 shows the relationship (overall and at each level of PEEP) between esophageal pressure swings at V_BEST_ and intrathoracic pressure with the two tested catheters. Esophageal pressure swings significantly correlate with intrathoracic pressure swings (Fig. 5). The overall difference between the measured esophageal swings and the intrathoracic swings was similar between the two catheters (*P* = 0.1651) and increased with PEEP (-0.64 ± 0.62, -0.07 ± 0.53, 1.43 ± 1.51, and 3.45 ± 3.94 at PEEP 0, 5, 10 and 15 cmH_2_O, respectively; *P* = 0.0197).

### Mechanical properties of the esophagus + esophageal balloon system did not change throughout the study

Additional balloon catheters volume-pressure curves, performed in absence of ventilation, were recorded in three pigs (Additional file 1, Fig. E19) at both the beginning and the end of the study. Esophagus + esophageal balloon elastance at the end of the study was not different from the one at the beginning (1.85 ± 0.60 *versus* 1.83 ± 0.59 cmH_2_O/mL; *P* = 0.750).

### Accuracy in estimating intrathoracic pressure inflating the esophageal balloon at V_producer_

Figures E20-E22 show the relationships (overall and at each level of PEEP) between esophageal pressure values (absolute and swings) measured at the volume suggested by the catheter’s producer with the two tested catheters, and intrathoracic pressure values. Both uncorrected and corrected esophageal pressure values were associated with intrathoracic pressure values but corrected esophageal pressure better approximated intrathoracic pressure (absolute values and swings, tables E25- E27). Correction significantly reduced the differences between esophageal pressure and intrathoracic pressure at different PEEP levels, however, its effect was influenced by the tested catheter (interaction term between catheter and correction was *P* < .0001 at end-expiration and *P* = 0.0021 at end-inspiration). We did not find statistically significant effects of PEEP and catheter on pressure swings. For further details, see Additional file 1.

## Discussion

The main findings of the present study in a swine model of eviscerated chest preserving the esophagus can be summarized as follows: 1. Uncorrected, directly measured, esophageal pressure provides a poor estimate of absolute values of intrathoracic pressure and the accuracy decreases at high levels of intrathoracic pressure; 2. Correction of esophageal balloon measurements based on the calculation of the esophagus + esophageal balloon elastance improved the estimation of absolute values of intrathoracic pressure; 3. Esophageal pressure swings accurately estimate intrathoracic pressure swings at low-medium intrathoracic pressures.

Transpulmonary pressure, computed as the difference between alveolar and pleural pressure, has been receiving particular interest in the recent years, since it represents the effective pressure across the lung parenchyma [6]. Since direct measurement of pleural pressure is challenging in the clinical setting, several techniques based on the measurement of the end-expiratory lung volume changes following a change in PEEP [25, 26] or esophageal manometry has been proposed to estimate pleural pressure, thus allowing the computation of transpulmonary pressure [6]. The most commonly applied technique uses the esophageal manometry. However, esophageal pressure depends on the mechanical properties of respiratory system, esophagus and esophageal catheter, thus raising concerns on the reliability of absolute esophageal pressure values as estimate of the corresponding pleural pressure [6, 7, 27, 28].

Furthermore, it has been shown the existence of a pleural pressure gradient with higher values in the dependent lung regions and lower values in the non-dependent regions, thus a single measured value of esophageal pressure cannot represent pleural pressure all over the entire chest, but it probably better reflects the value surrounding the esophagus [14–16].

Esophageal pressure swings instead showed to be similar to intrathoracic pressure swings [27, 28]; for this reason, some authors suggested to compute the transpulmonary pressure by calculating the partitioned respiratory mechanics (lung and chest compliance) through the esophageal pressure swings [7, 15]. Furthermore, the Baydur test has been developed as tool to assess the correct placement of the balloon and to optimize the esophageal balloon inflation volume, thus improving the accuracy of esophageal pressure swings in estimating the intrathoracic swings.

More recently, some authors proposed to compute the transpulmonary pressure by subtracting the measured absolute value of esophageal pressure from the alveolar pressure and suggested to set positive end-expiratory pressure to obtain slightly positive end-expiratory transpulmonary pressure [8, 9]. Furthermore, multiple correction techniques aiming to also increasing the reliability of the absolute values of esophageal pressure have been proposed. In particular, Mojoli et al. introduced a technique based on the identification of V_BEST_ (defined as the esophageal balloon inflation volume that maximizes the respiratory swing of esophageal pressure) and on calculation of the esophageal wall + esophageal balloon elastance, with subsequent improved estimation of absolute values and swings of intrathoracic pressure [22].

However, few data on comparison between esophageal pressure and real intrathoracic pressure are available, mainly due to the technical difficulties to measure intrathoracic pressure even in experimental models [15, 29].

In this study we aimed to clarify the accuracy of esophageal pressure as estimate of intrathoracic pressure by developing an ex vivo swine model enrolling animals previously included in another study on carbon-dioxide removal to minimize the number of animals sacrificed for scientific aims. Previous studies verified the accuracy of esophageal balloons simulating the pleural cavity, without esophagus, with in vitro models characterized by air-tight [20], glass [30] or rigid plastic chambers [19] or glass chambers including a rubber test lungs [24]. Another group [31] developed a biomechanical model of the thoracic cavity using a temperature- and pressure-controlled 2.5-L polycarbonate box mounting at the extremities an ex vivo pig esophagus. The esophagi were harvested from pigs and preserved using a solution for several days to maintain tissue elasticity, prevent tissue desiccation, and inhibit mold or bacterial growth. The model intentionally excluded ventilatory tidal displacements. We conducted our experiments on a mechanically ventilated ex vivo swine eviscerated chest, preserving the esophagus. The present study begun immediately after the swine sacrifice to maintain both the biomechanical characteristics of the tissues and pressure transmission through the esophageal wall as close as possible to the physiological ones. We verified that, despite the absence of perfusion, esophageal mechanical characteristics were not modified throughout the whole study. Our ex vivo model was developed to obtain an easily and accurately measurable absolute intrathoracic pressure value. The addition of the inelastic plastic bag positioned into the cavity to avoid air leaks did not influence the measurements as the plastic bag resting volume was more than double than the intrathoracic volume at maximal intrathoracic pressure, thus the plastic bag never stretched. Moreover, the removal of the organs from the thoracic cavity was performed to overcome the challenge related to the pleural pressure gradient by having a single homogenous intrathoracic pressure. Finally, our model allowed an accurate measurement of chest wall compliance.

We compared the esophageal pressure with the intrathoracic pressure, and we showed that uncorrected esophageal pressure poorly estimates the corresponding intrathoracic value. Esophageal swings instead better represent the corresponding intrathoracic swings, but the accuracy decreased at high level of intrathoracic pressure, particularly when end-expiratory intrathoracic pressure was 15 cmH_2_O. However, due to the absence of lungs, PEEP levels used in our ex vivo model generated considerably higher intrathoracic pressures compared to what would be observed with the same PEEP in patients with lung injury.

In this model, the application of the correction technique taking into account the mechanical response of esophageal wall to the balloon inflation, greatly improved the accuracy of the measurement, thus providing more reliable estimates of absolute values of intrathoracic pressure.

In the study we tested two different esophageal balloons, and we obtained better results with the Nutrivent, probably due to the larger resting balloon volume. Notably the esophageal wall + esophageal balloon elastance resulted lower with the Nutrivent catheter compared to the Cooper suggesting that the Nutrivent catheter has probably better mechanical properties or works in a more favorable part of the esophageal volume-pressure curve.

The described correction technique considering the esophageal wall + esophageal balloon elastance could be also applied in patients, thus allowing increased accuracy in estimating pleural pressure in the regions surrounding the esophagus. However, we found that V_BEST_ was highly variable among different PEEP and catheters (see Additional file 1, Tables E5-E6). Mojoli et. al found that V_BEST_ varied from 0.5 to 6 mL in their series of sedated and paralyzed patients with acute respiratory failure and under pressure-controlled mechanical ventilation [22]. These results suggest that the optimal esophageal catheter filling volume should be determined before every measurement as it is specific to the intrathoracic pressure of any given subject and disease state. Indeed, it should be remembered that intrathoracic pressure is not homogenous in the chest and its gradient is even more variable in the subjects with acute respiratory failure. Moreover, also inspiratory and expiratory breathing efforts affect the inhomogeneity of pleural pressure. In absence of correction, esophageal pressure overestimates intrathoracic pressure and the overestimation increased at high levels of intrathoracic pressure. Our data strongly support that, if absolute values of intrathoracic pressure are required to guide mechanical ventilation, uncorrected absolute end-expiratory and end-inspiratory values should not be used since they unacceptably overestimate the real intrathoracic absolute values and result in potential harm for the patient. The overestimation is affected by both the esophageal balloon inflation volume and intrathoracic pressure value; thus, the correction factor should be personalized on both the subject and on the setting. The described correction method proved to be reliable in providing absolute values that could be more effectively used in clinical practice [32]. Our main findings are based on esophageal pressures measured at V_BEST_ but we found that they can be extended to pressures measured inflating the esophageal balloon at the volume recommended by the catheter’s producer. Inflating the balloon at V_producer_ will slightly simplify the suggested correction procedure eliminating the need for inflation VP curve. However, while performing individual VP curves, attention should be paid to the position of the inflating volume of the balloon within the quasi-linear part of the curve.

The study aims on clarifying the relationship between esophageal and intrathoracic pressures in a model where intrathoracic pressure can be easily and accurately measured. Although titration of mechanical ventilation (e.g., setting of positive end-expiratory pressure and end-inspiratory transpulmonary pressure) is beyond the aim of this study, we believe that our data provide the rationale to design further studies on humans to develop more adequate and effective strategies to optimize and individualize ventilatory parameters. Furthermore, we believe that both our model and our findings could be used to develop new studies to improve our knowledge on respiratory physiology, especially chest wall mechanics.

### Limitations

The study presents some limitations. First, it was performed in an ex vivo model of eviscerated chest because an in vivo model would have required artificial heart and lung support with extracorporeal circulation and subsequent decreased feasibility. Second, the described model was developed after multiple attempts due to air leak and development of subcutaneous emphysema, thus we needed to replace the parietal pleura with an inelastic plastic bag. However, since the bag was inelastic and had a resting volume more than double the chest volume at maximum intrathoracic pressure, it had no role in the evaluation of the correction technique on the quality of the relationship between esophageal and intrathoracic pressures. The bag did not modify also the absolute value of intrathoracic pressure, and therefore, the chest wall compliance.

Third, ischemia of the esophagus could have altered its mechanical properties although we did not observe differences in esophagus-balloon elastance, similar to values reported on humans [22], at the end and at the beginning of the study. Fourth, according to the 3Rs principle, the study was conducted at the end of a 24-h study evaluating a low-flow extracorporeal carbon dioxide removal technique without, in our opinion, affecting the results. Fifth, the obtained results seem solid and reliable although the sample size is small.

We think that the proposed eviscerated chest model could be used for future studies on mechanical properties of the chest wall.

## Conclusions

In this study conducted on swine model of eviscerated chest, preserving esophagus, uncorrected esophageal pressure poorly estimates absolute values of intrathoracic pressure, particularly at high levels of intrathoracic pressure, while correction increased the accuracy of the estimation. Esophageal pressure swings accurately estimate intrathoracic pressure swings at low-medium intrathoracic pressures.

## Supplementary Information

Additional file 1. Additional materials and methods and results.

## Data Availability

The datasets used and/or analysed during the current study are available from the corresponding author on reasonable request.

## Abbreviations

E_ES_: Elastance of the esophagus, computed according to Eq. 3 (see Additional file 1—materials and methods)
PEEP: Positive end expiratory pressure
V_BEST_: The optimal catheter filling volume
V_MAX_: The upper limit of quasi-linear portion of the end-expiratory VP curve, it represents the larger filling volume that does not induce overstretch of the balloon wall
V_MIN_: The lower limit of quasi-linear portion of the end-expiratory VP curve, it represents the smallest filling volume to pressurize the catheter at the same pressure surrounding the esophageal balloon
VP curves: Volume-pressure curves
V_producer_: The filling volume recommended by the catheter’s producer
