# Supplementary material for: Esophageal pressure as estimation of pleural pressure: a study in a model of eviscerated chest
Source: BMC Anesthesiol. 2024 Nov 14;24:415. doi: 10.1186/s12871-024-02806-0 (PMC11562330; doi:10.1186/s12871-024-02806-0)
Supplement: Supplementary file 1 — Additional file 1. Additional materials and methods and results. [file 12871_2024_2806_MOESM1_ESM.docx]

**Esophageal pressure as estimation of pleural pressure: a study in a model of eviscerated chest**

Gaetano Florio*, Eleonora Carlesso*, Francesco Mojoli, Fabiana Madotto, Luigi Vivona, Chiara Minaudo, Michele Battistin, Sebastiano M. Colombo, Stefano Gatti, Simone Sosio, Antonio Pesenti, Giacomo Grasselli, Alberto Zanella.

**Online Data Supplement**

INDEX

[ADDITIONAL MATERIALS AND METHODS 3](#_Toc179375119)

[STUDY DESIGN 3](#_Toc179375120)

[COMPUTATIONS 4](#_Toc179375121)

[COMPARISON BETWEEN ESOPHAGEAL PRESSURE MEASURED AT PRODUCER BALLOON INFLATION VOLUME AND INTRATHORACIC PRESSURE 4](#_Toc179375122)

[BALLOON FILLING VOLUME SELECTION (V_BEST_) 4](#_Toc179375123)

[CORRECTION 6](#_Toc179375124)

[EXAMPLE OF VBEST SELECTION AND OF ESOPHAGEAL PRESSURE VALUES CORRECTION 6](#_Toc179375125)

[COMPARISON BETWEEN ESOPHAGEAL PRESSURE (MEASURED AT V_BEST_ and at PRODUCER VOLUME) AND INTRATHORACIC PRESSURE. EFFECT OF CORRECTION. 9](#_Toc179375126)

[COMPARISON BETWEEN ESOPHAGUS-ESOPHAGEAL BALLOON ELASTANCES AT THE BEGINNING AND AT THE END OF THE STUDY. 9](#_Toc179375127)

[ADDITIONAL RESULTS 10](#_Toc179375128)

[Chest wall compliance 10](#_Toc179375129)

[Average esophageal balloon volume-pressure curves 11](#_Toc179375130)

[Figure E6-Figure E15. Esophageal balloon volume-pressure curves fittings 13](#_Toc179375131)

[TABLE E1-E2. Parameters of end-expiratory sigmoidal curve, according to catheter. 23](#_Toc179375132)

[TABLE E3-E4. Parameters of end-inspiratory sigmoidal curve, according to catheter. 24](#_Toc179375133)

[TABLE E5-E6. Parameters estimated during the CORRECTION, according to catheter. 25](#_Toc179375134)

[TABLE E7-E16. LINEAR REGRESSIONS RESULTS 28](#_Toc179375135)

[Table E17-E19. Estimated parameters of each linear regression model and comparison with those of identity line (intercept=0 and regression coefficient =1) (F test), according to pressures. 33](#_Toc179375136)

[MODEL SELECTION 36](#_Toc179375137)

[RESULTS OF GENERAL LINEAR MIXED MODELS APPLIED TO END-EXPIRATORY PRESSURE DURING MANUAL INCREMENTAL STEP INFLATION 38](#_Toc179375138)

[RESULTS OF GENERAL LINEAR MIXED MODELS APPLIED TO END-INSPIRATORY PRESSURE DURING MANUAL INCREMENTAL STEP INFLATION 40](#_Toc179375139)

[RESULTS OF GENERAL LINEAR MIXED MODELS APPLIED TO PRESSURE SWINGS DURING MANUAL INCREMENTAL STEP INFLATION 42](#_Toc179375140)

[COMPARISON BETWEEN ESOPHAGUS-ESOPHAGEAL BALLOON ELASTANCES AT THE BEGINNING AND AT THE END OF THE STUDY 44](#_Toc179375141)

[PRODUCER VOLUME 45](#_Toc179375142)

[MODEL SELECTION 45](#_Toc179375143)

[RESULTS OF GENERAL LINEAR MIXED MODELS APPLIED TO END-EXPIRATORY PRESSURE DURING MANUAL INCREMENTAL STEP INFLATION - Esophageal balloon inflated at v_producer_ 46](#_Toc179375144)

[RESULTS OF GENERAL LINEAR MIXED MODELS APPLIED TO END-INSPIRATORY PRESSURE DURING MANUAL INCREMENTAL STEP INFLATION - Esophageal balloon inflated at v_producer_ 48](#_Toc179375145)

[RESULTS OF GENERAL LINEAR MIXED MODELS APPLIED TO PRESSURE SWINGS DURING MANUAL INCREMENTAL STEP INFLATION - Esophageal balloon inflated at v_producer_ 50](#_Toc179375146)

##

## ADDITIONAL MATERIALS AND METHODS

### STUDY DESIGN

**Figure E1:** *figure represents the study design. All 5 pigs underwent 8 combinations of catheter (2) and PEEP values (4) applied in random order.*

CATHETER

(RANDOM ORDER):

- COOPER
- NUTRIVENT

PEEP

(RANDOM ORDER):

- 0 cmH_2_O
- 5 cmH_2_O
- 10 cmH_2_O
- 15 cmH_2_O

### COMPUTATIONS

Chest-wall compliance was computed as the ratio of tidal volume to the difference of end-inspiratory and end-expiratory intrathoracic pressures.

Swings were defined as differences between end-inspiratory and end-expiratory pressures.

### COMPARISON BETWEEN ESOPHAGEAL PRESSURE MEASURED AT PRODUCER BALLOON INFLATION VOLUME AND INTRATHORACIC PRESSURE

Intrathoracic pressure and esophageal pressure (end-expiratory, end-inspiratory and swings) measured at producer balloon inflation volume were compared by generalized linear mixed models. The models included the type of pressure measurement (intrathoracic or esophageal), PEEP and their interaction as fixed effects. Intercept, PEEP and type of measurements were included as random effects at pig level. Compound symmetry covariance structure was used. Tests were two-sided and P-value <0.05 was considered significant. Multiple comparisons were adjusted with Bonferroni’s correction.

### BALLOON FILLING VOLUME SELECTION (V_BEST_)

The appropriate balloon filling volumes of the 2 catheters tested were identified according to a selection technique proposed by Mojoli et al. (CRITICAL CARE 2016; 20:98). Accordingly, the optimal working range of the balloon is the quasi-linear section of the end-expiratory relationships between balloon filling volume and esophageal pressure (VP curve). This section was delimited by V_MIN_, the smallest filling volume to pressurize the catheter at the same pressure surrounding the esophageal balloon, and V_MAX_, the larger filling volume that does not induce overstretch of the balloon wall.

To optimize the selection of V_MIN_ and V_MAX_ on the expiratory curves and to reduce interobserver/intraobserver variability, we fitted the experimental data points with a sigmoid equation introduced by Harris et al. to curve-fit volume-pressure curve data sets of respiratory system (AM J RESPIR CRIT CARE MED 2000;161:432–439; Intensive Care Med Exp 2017;5(1):35) and then we computed objective parameters:

$V =a+ b/\left( 1+ e^{{-\left( P-c \right)}/d} \right)$ (eq. 1)

Where V represents volume, P represents pressure, **a**, represents the lower asymptote of balloon filling volume; **b** represents the distance from a to the upper asymptote of balloon filling volume; **c** represents the pressure of the true inflection point of the curve (where concavity changes direction); **d** represents the distance from **c** of the zone of high slope of the curve.

**Figure E2:** *Figure shows an example of the sigmoidal curve drawn with the equation of Harris et al. The solid line represents the equation computed using the following parameter values: a=0, b=8000, c=5, d=1. Dashed lines represents the values of the curve parameters. Green vertical dashed lines represents the quasi-linear portion of the curve. See text for further description.*

To comply with the model of Harris et al. data were fitted using pressure as independent variable. The equation was fitted to the experimental data using the Marquardt iterative algorithm to minimize the sum of squared function values. The algorithm was set to run until the convergence was met (relative offset measure of Bates and Watts > 10^-5^ in a maximum of 2000 iterations). The initial guess coefficients were: **a**=minimum volume; **b**=maximum volume - minimum volume; **c** ranged from minimum esophageal pressure + (maximum esophageal pressure- minimum esophageal pressure)/2 to maximum esophageal pressure; **d**= (maximum esophageal pressure- minimum esophageal pressure)/4.

The quasi-linear section of the sigmoid curve was delimited by the pressure values at the point of maximum slope increase (P_MIN_=c-1.317×d) and maximum slope decrease (P_MAX_=c+1.317×d). The corresponding balloon filling volumes, V_MIN_ and V_MAX_, were computed substituting P_MIN_ and P_MAX_, respectively, into equation 1.

The appropriate catheter filling volume (V_BEST_) to measure esophageal pressure was identified within the quasi-linear range of the curve as the volume providing the maximum difference between end-inspiratory and end-expiratory esophageal pressures. In order to compute this difference, we also fitted the inspiratory curve with a sigmoidal equation and a procedure similar to the one applied for the expiratory limb of the curve.

### CORRECTION

The end-expiratory and end-inspiratory esophageal pressures obtained at V_BEST_ were then corrected subtracting the artifactual increase of pressure generated by the esophagus wall (P_EW_) due to the balloon filling and calculated as:

P_EW_ = (V_X_ – V_MIN_) * E_ES_ (eq. 2)

where V_X_ was any filling volume above V_MIN_ and E_ES_ was the slope of the quasi-linear section of the expiratory curve, which was considered the elastance of the system esophagus + esophageal balloon and was computed as the ratio of (P_MAX_-P_MIN_) to (V_MAX_-V_MIN_).

E_ES_ = (P_MAX_-P_MIN_) / (V_MAX_-V_MIN_) (eq. 3)

The same correction procedure was also applied to end-expiratory and end-inspiratory esophageal pressures obtained at the balloon filling volume suggested by the producer (4 ml for Nutrivent, 1 ml for Cooper).

The same P_EW_ correction factor was applied to correct both the end-expiratory and end-inspiratory esophageal pressure values.

### EXAMPLE OF VBEST SELECTION AND OF ESOPHAGEAL PRESSURE VALUES CORRECTION


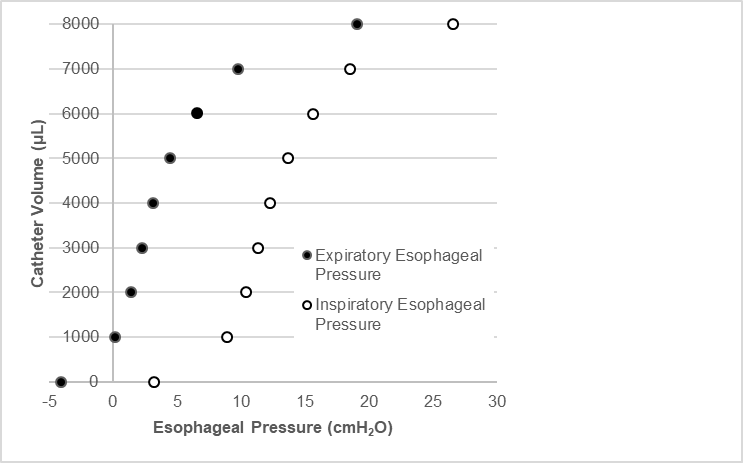
Experimental data points of the esophageal balloon volume-pressure curves at PEEP 0 cmH_2_O measured during the application of manual incremental step inflation procedure with *Nutrivent* catheter for pig n°4.

| **Catheter Volume**  **(μL)** | **Espiratory Esophageal Pressure**  **(cmH_2_O)** | **Inspiratory Esophageal Pressure**  **(cmH_2_O)** |
| --- | --- | --- |
| 0 | -4.087 | 3.1998 |
| 1000 | 0.1462 | 8.8957 |
| 2000 | 1.3992 | 10.4103 |
| 3000 | 2.2263 | 11.2967 |
| 4000 | 3.156 | 12.2413 |
| 5000 | 4.4523 | 13.621 |
| 6000 | 6.6022 | 15.6205 |
| 7000 | 9.7769 | 18.502 |
| 8000 | 19.0068 | 26.5029 |

End-expiratory and end-inspiratory experimental data points were fitted with a sigmoid equation (equation 1). Analyses were performed using NLIN procedure in SAS software v.9.4 (SAS Institute, Cary, NC, USA).

The following table reports the obtained equation parameters and R^2^ for expiratory and inspiratory curves, respectively:

|  | **a**  **(μL)** | **b**  **(μL)** | **c**  **(cmH_2_O)** | **d**  **(cmH_2_O)** | **R^2^** |
| --- | --- | --- | --- | --- | --- |
| **Expiratory curve** | -538.33 | 8250.3 | 3.023 | 2.3204 | 0.99106 |
| **Inspiratory curve** | -307.74 | 8082.29 | 12.271 | 2.3412 | 0.99335 |


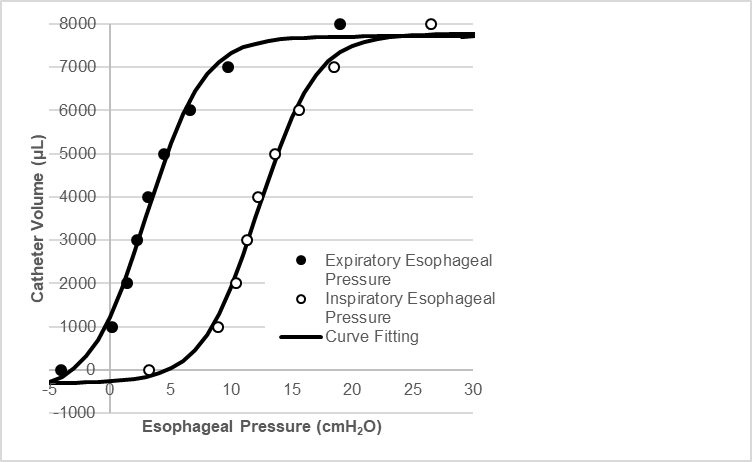


Accordingly, the quasi-linear part of the **expiratory** curve was delimited by the pressure values at the point of maximum slope increase P_MIN_ = c – 1.327 x d = -0.0329668 cmH_2_O and maximum slope decrease P_MAX_ = 6.0789668 cmH_2_O and the corresponding balloon filling volumes can be computed substituting P_MIN_ and P_MAX_ into equation 1 and a, b, c, d with the parameters of the expiratory curve: V_MIN_ = 1205.106 μL and V_MAX_ = 5968.534 μL.

The balloon filling volume was the independent variable of the analysis. Accordingly, equation 1 should be most properly written as:

$$c-d\times\ln\frac{b-V+a}{V-a}$$

and represented inverting the axes.


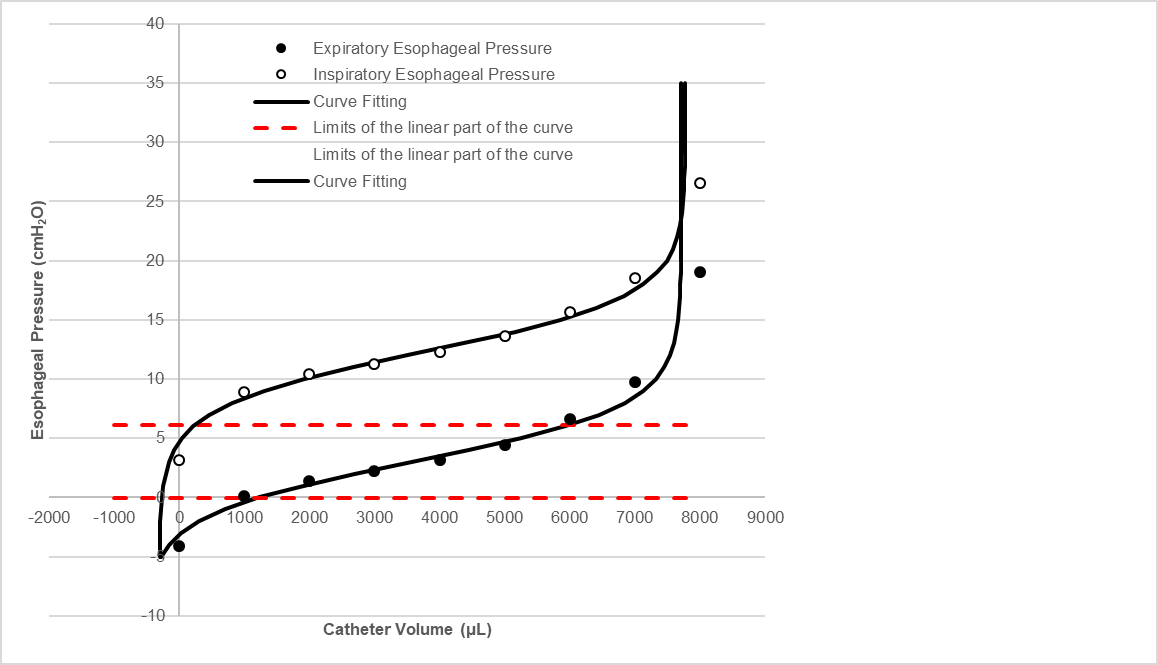


We then estimated end-inspiratory and end-expiratory esophageal pressures at varying catheter filling volumes (Vx) within the range [VMIN, VMAX] and then we computed the end-inspiratory-to-end-expiratory pressure difference (dP) at Vx:

dP = (end-inspiratory pressure)_Vx_ – (end-expiratory pressure)_Vx_

The appropriate catheter filling volume (V_BEST_) to measure esophageal pressure was identified as the volume providing the maximum dP value. Our example provided a maximum dP value equal to 9.1122 cmH_2_O at an end-expiratory pressure value = 5.2671 cmH_2_O and an end-inspiratory pressure value = 14.3793 cmH_2_O. V_BEST_ was equal to 5439.3 μL.


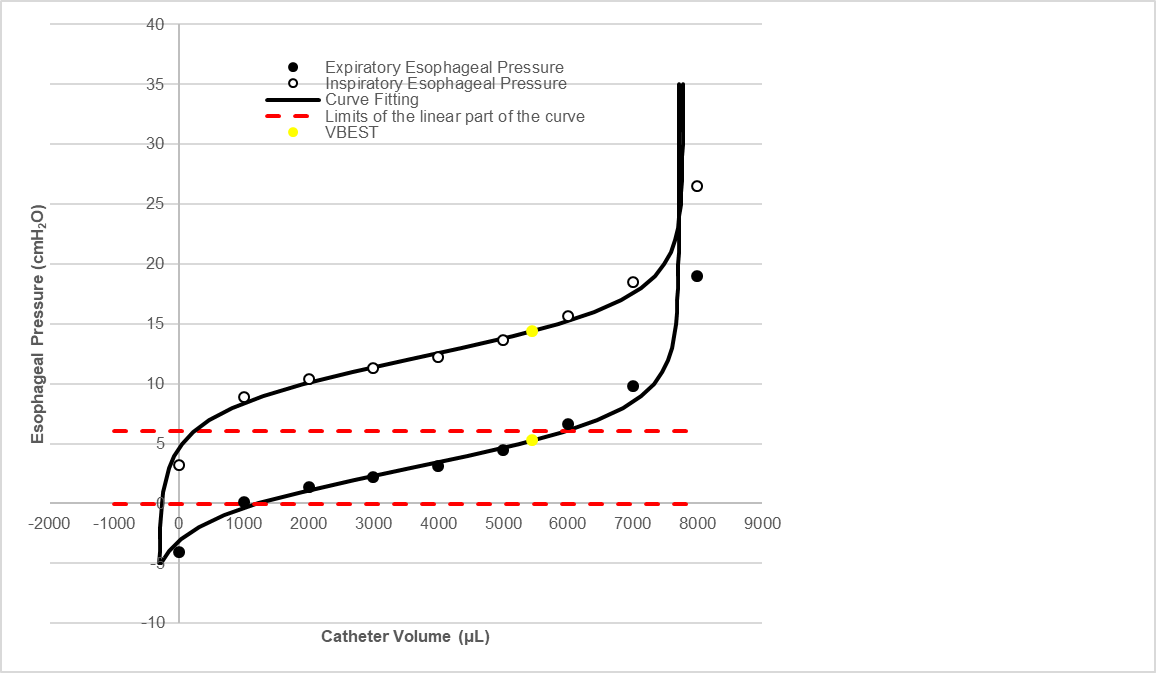


According to equation 3, the elastance of the system esophagus + esophageal balloon was computed as:

E_ES_ = (P_MAX_-P_MIN_) / (V_MAX_-V_MIN_) = 0.001282506 cmH_2_O/μL = 1.282506 cmH₂O/mL

The artifactual increase of pressure generated by the esophagus wall (P_EW_) due to the balloon filling was calculated, according to equation 2, as:

P_EW_ = (VBEST – V_MIN_) * E_ES_ = 5.4304 cmH_2_O

Finally, the corrected end-expiratory esophageal pressure at V_BEST_ was equal to:

end-expiratory esophageal pressure - P_EW_ = 5.2671 - 5.4304 = **-0.1633 cmH_2_O**

while the corrected end-inspiratory esophageal pressure at V_BEST_ was equal to:

end-inspiratory esophageal pressure - P_EW_ = 14.3793 - 5.4304 = **8.9489 cmH_2_O**

The corresponding intrathoracic pressure values measured were equal to **0.0413 cmH_2_O** at end expiration and **9.4249 cmH_2_O** at end inspiration.

### COMPARISON BETWEEN ESOPHAGEAL PRESSURE (MEASURED AT V_BEST_ and at PRODUCER VOLUME) AND INTRATHORACIC PRESSURE. EFFECT OF CORRECTION.

Descriptive statistics of continuous variables were reported using mean (± standard deviation (SD)). The relationships between intrathoracic pressures and esophageal pressures (end-expiratory, end-inspiratory and swings) were modeled by univariate linear regressions according to the catheter, for uncorrected and corrected values and also according to PEEP levels (0, 5, 10 and 15 cmH_2_O). Estimated parameters of each linear regression model were compared with those of identity line (intercept=0 and regression coefficient =1) using F test.

Generalized linear mixed models with fixed and random effects (including intercept) were applied to test the effects of catheter, PEEP and correction (as appropriate) on the difference between intrathoracic and esophageal pressures at end-expiration, end-inspiration and swings. Inclusion of interaction terms between fixed effects was based on Akaike information criterion (AIC) and on the parsimony principle for a statistical model. Compound symmetry covariance structure was used. Pairwise comparisons were tested with Bonferroni’s correction for multiple testing.

### COMPARISON BETWEEN ESOPHAGUS-ESOPHAGEAL BALLOON ELASTANCES AT THE BEGINNING AND AT THE END OF THE STUDY.

Esophagus+esophageal balloon volume-pressure curves at 0 cmH_2_O PEEP measured with Cooper catheter for pig n°6 and 8 and Nutrivent catheter for pig n°7 were measured at the beginning (START) and at the end (END) of the study to compare esophagus+esophageal balloon elastance. Curves were fitted according to equation 1 and the quasi-linear part of the curves were delimited as described above. Finally, elastance values were computed according to equation 3. Wilcoxon Signed Rank Test was used to compare mean values observed at the beginning and the end of the study.

All statistical tests of significance were two sided (α of 0.05). Analyses were performed using SAS software v.9.4 (SAS Institute, Cary, NC, USA) and SigmaPlot (Systat Software, Inc, San Jose, CA).

## ADDITIONAL RESULTS

### Chest wall compliance

**Figure E3:** *Figure represents the chest wall compliance, at different PEEP levels, computed as the ratio of tidal volume to the difference of end-inspiratory and end-expiratory intrathoracic pressures (intrathoracic pressures measured while testing both Cooper and Nutrivent catheters).*

*PEEP effect was statistically significant (P<0.0001). *** P<0.001 vs PEEP 0; # P<0.05 vs PEEP 5; ### P<0.001 vs PEEP 5; §§ P<0.01 vs PEEP 10.*

### Average esophageal balloon volume-pressure curves

**Figure E4. Average volume-pressure curves with Cooper catheter**

*Figure represents the average esophageal balloon volume-pressure curves at different PEEP levels (0, 5, 10, 15 cmH_2_O) measured with Cooper catheter. Dots represent mean ± standard deviations of experimental data points at end-inspiration (white) and end-expiration (black). Dotted line with green band represents mean ± standard deviations of intrathoracic end-inspiratory pressure while dotted line with yellow band represents mean ± standard deviations of intrathoracic end-expiratory pressure*

**Figure E5. Average volume-pressure curves with Nutrivent catheter**

*Figure represents the average esophageal balloon volume-pressure curves at different PEEP levels (0, 5, 10, 15 cmH_2_O) measured with Nutrivent catheter. Dots represent mean ± standard deviations of experimental data points at end-inspiration (white) and end-expiration (black). Dotted line with green band represents mean ± standard deviations of intrathoracic end-inspiratory pressure while dotted line with yellow band represents mean ± standard deviations of intrathoracic end-expiratory pressure*

### Figure E6-Figure E15. Esophageal balloon volume-pressure curves fittings

**Figure E6:** *figure represents the esophageal balloon volume-pressure curves fittings at different PEEP levels (0, 5, 10, 15 cmH_2_O) measured during the application of manual incremental step inflation procedure with Cooper catheter for pig n°4. Dots represent experimental data points at end-inspiration (white), end-expiration (black), end-inspiratory V_BEST_ (yellow), end-inspiratory V_BEST_ (green). Continuous black lines represent fittings of the experimental data. Dashed red lines represent the limits of the quasi-linear part of the end-expiratory curve.*


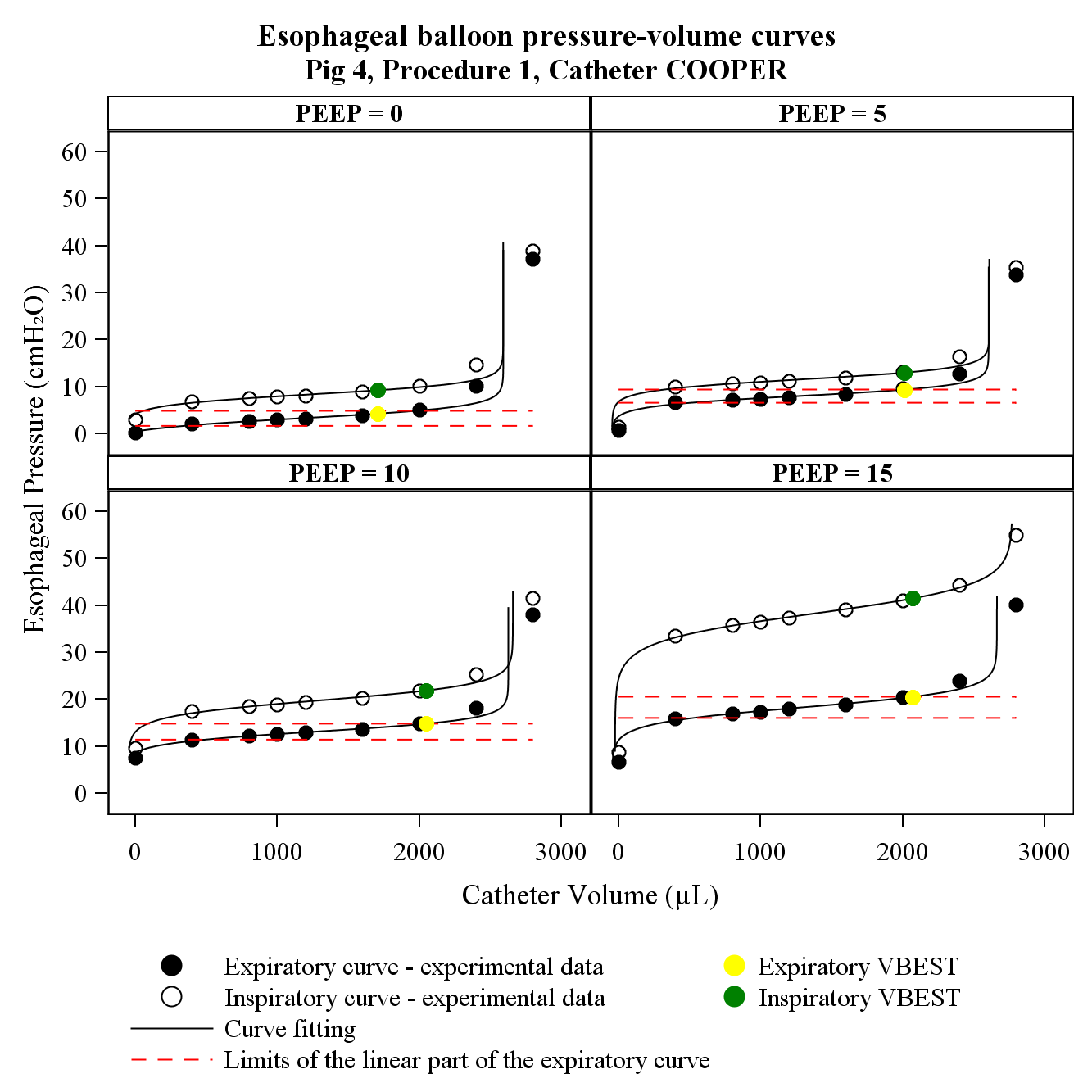


Limits of the quasi-linear part of the expiratory curve

**Figure E7:** *figure represents the esophageal balloon volume-pressure curves fittings at different PEEP levels (0, 5, 10, 15 cmH_2_O) measured during the application of manual incremental step inflation procedure with Cooper catheter for pig n°5. Dots represent experimental data points at end-inspiration (white), end-expiration (black), end-inspiratory V_BEST_ (yellow), end-inspiratory V_BEST_ (green). Continuous black lines represent fittings of the experimental data. Dashed red lines represent the limits of the quasi-linear part of the end-expiratory curve.*


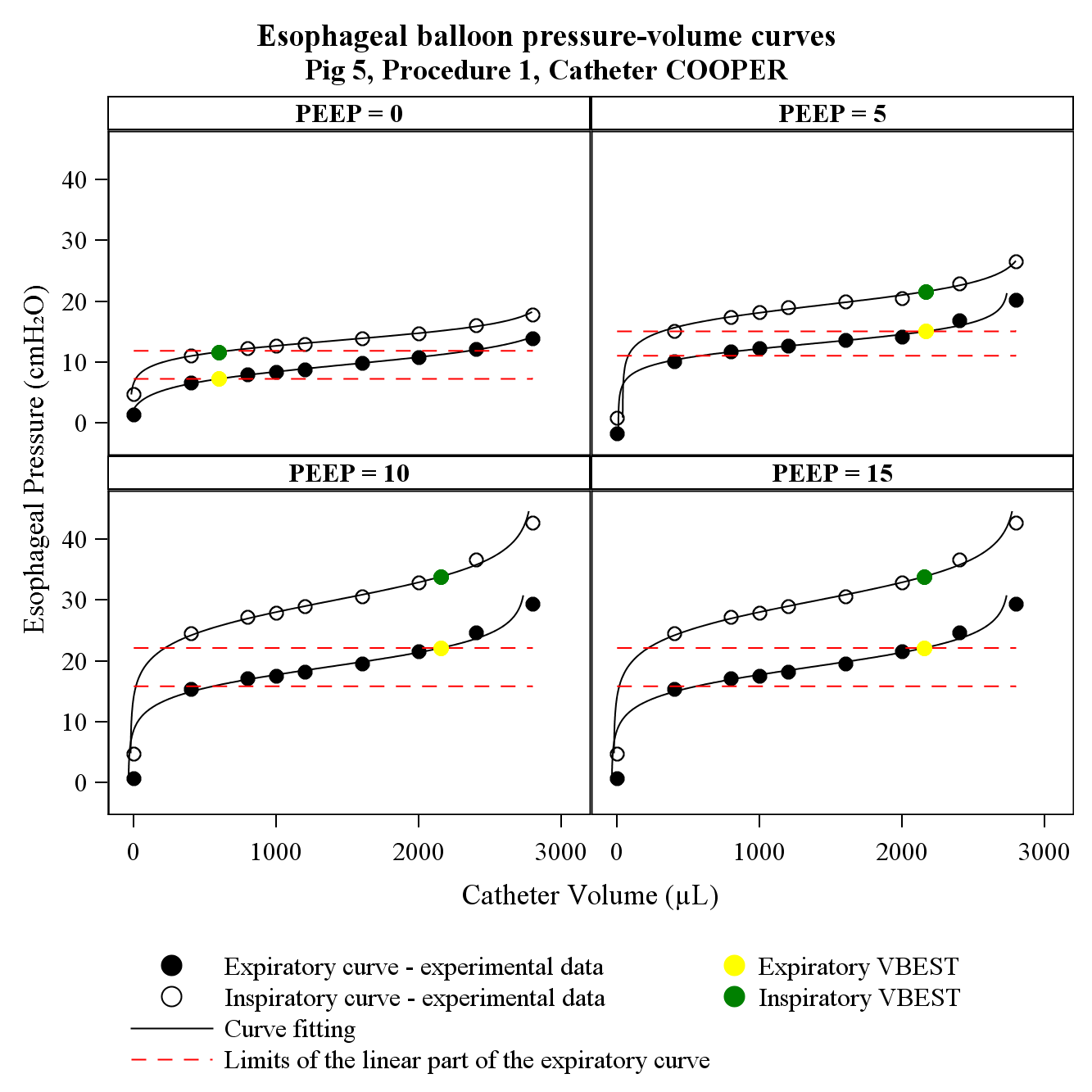


Limits of the quasi-linear part of the expiratory curve

**Figure E8:** *figure represents the esophageal balloon volume-pressure curves fittings at different PEEP levels (0, 5, 10, 15 cmH_2_O) measured during the application of manual incremental step inflation procedure with Cooper catheter for pig n°6. Dots represent experimental data points at end-inspiration (white), end-expiration (black), end-inspiratory V_BEST_ (yellow), end-inspiratory V_BEST_ (green). Continuous black lines represent fittings of the experimental data. Dashed red lines represent the limits of the quasi-linear part of the end-expiratory curve.*


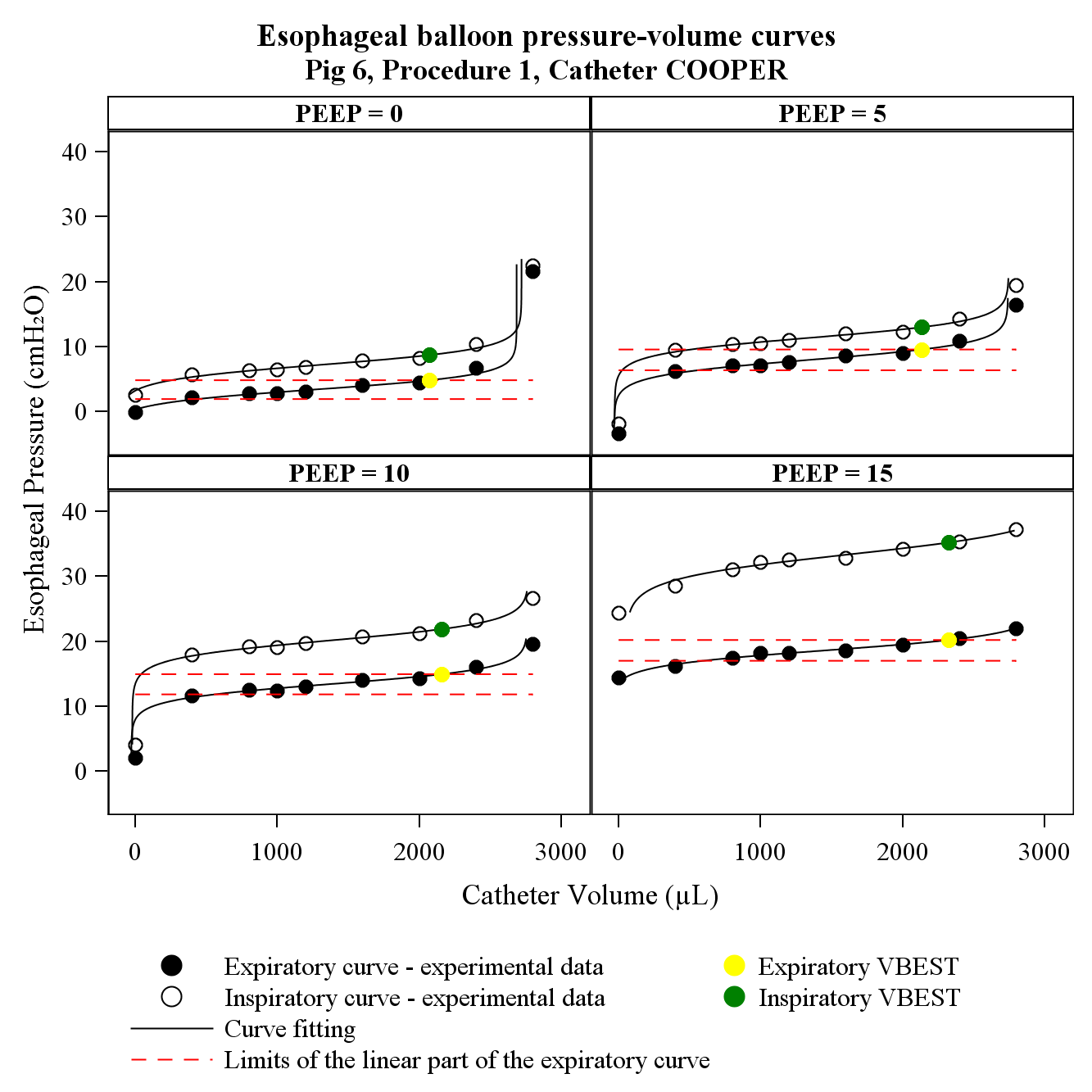


Limits of the quasi-linear part of the expiratory curve

**Figure E9:** *figure represents the esophageal balloon volume-pressure curves fittings at different PEEP levels (0, 5, 10, 15 cmH_2_O) measured during the application of manual incremental step inflation procedure with Cooper catheter for pig n°7. Dots represent experimental data points at end-inspiration (white), end-expiration (black), end-inspiratory V_BEST_ (yellow), end-inspiratory V_BEST_ (green). Continuous black lines represent fittings of the experimental data. Dashed red lines represent the limits of the quasi-linear part of the end-expiratory curve.*


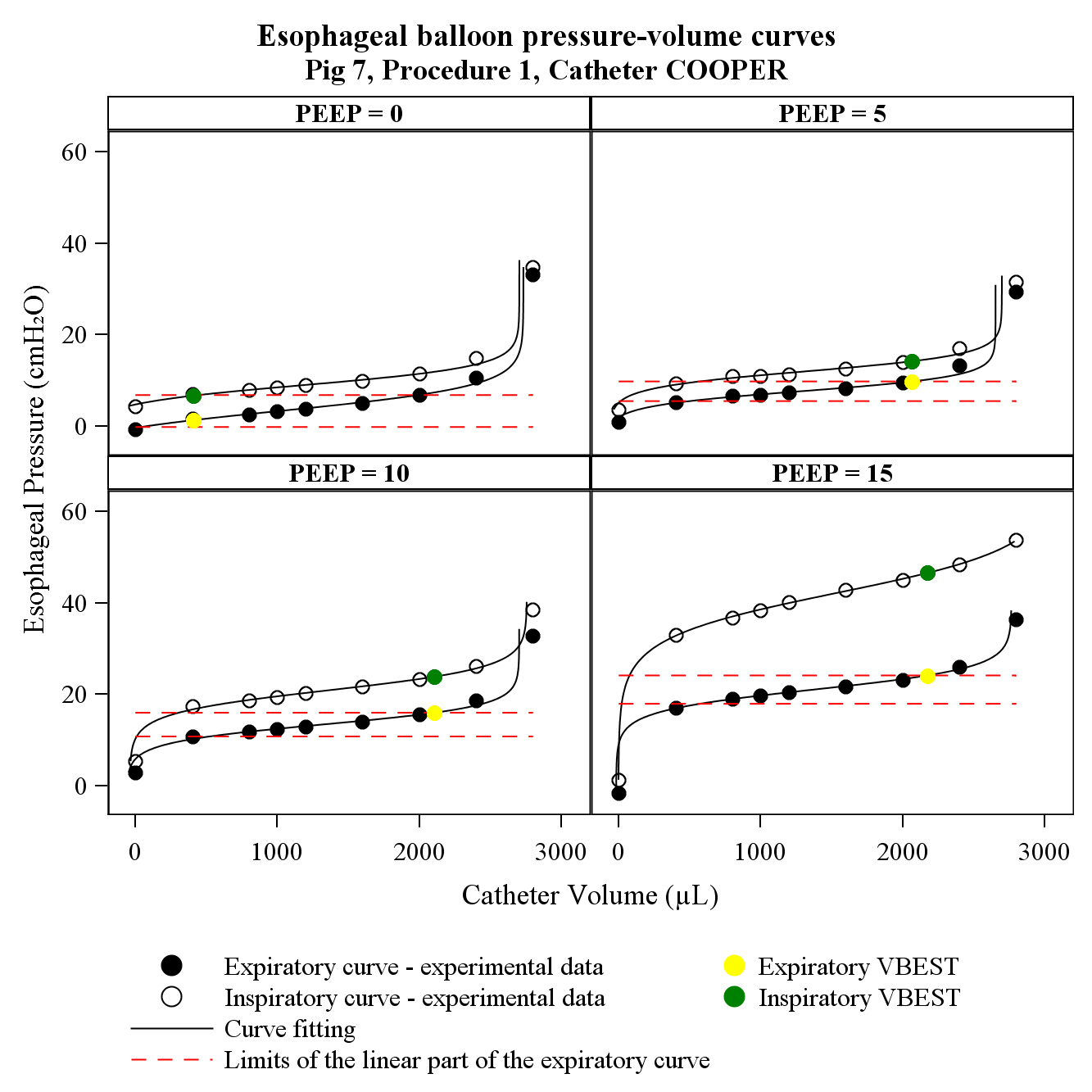


Limits of the quasi-linear part of the expiratory curve

**Figure E10:** *figure represents the esophageal balloon volume-pressure curves fittings at different PEEP levels (0, 5, 10, 15 cmH_2_O) measured during the application of manual incremental step inflation procedure with Cooper catheter for pig n°8. Dots represent experimental data points at end-inspiration (white), end-expiration (black), end-inspiratory V_BEST_ (yellow), end-inspiratory V_BEST_ (green). Continuous black lines represent fittings of the experimental data. Dashed red lines represent the limits of the quasi-linear part of the end-expiratory curve.*


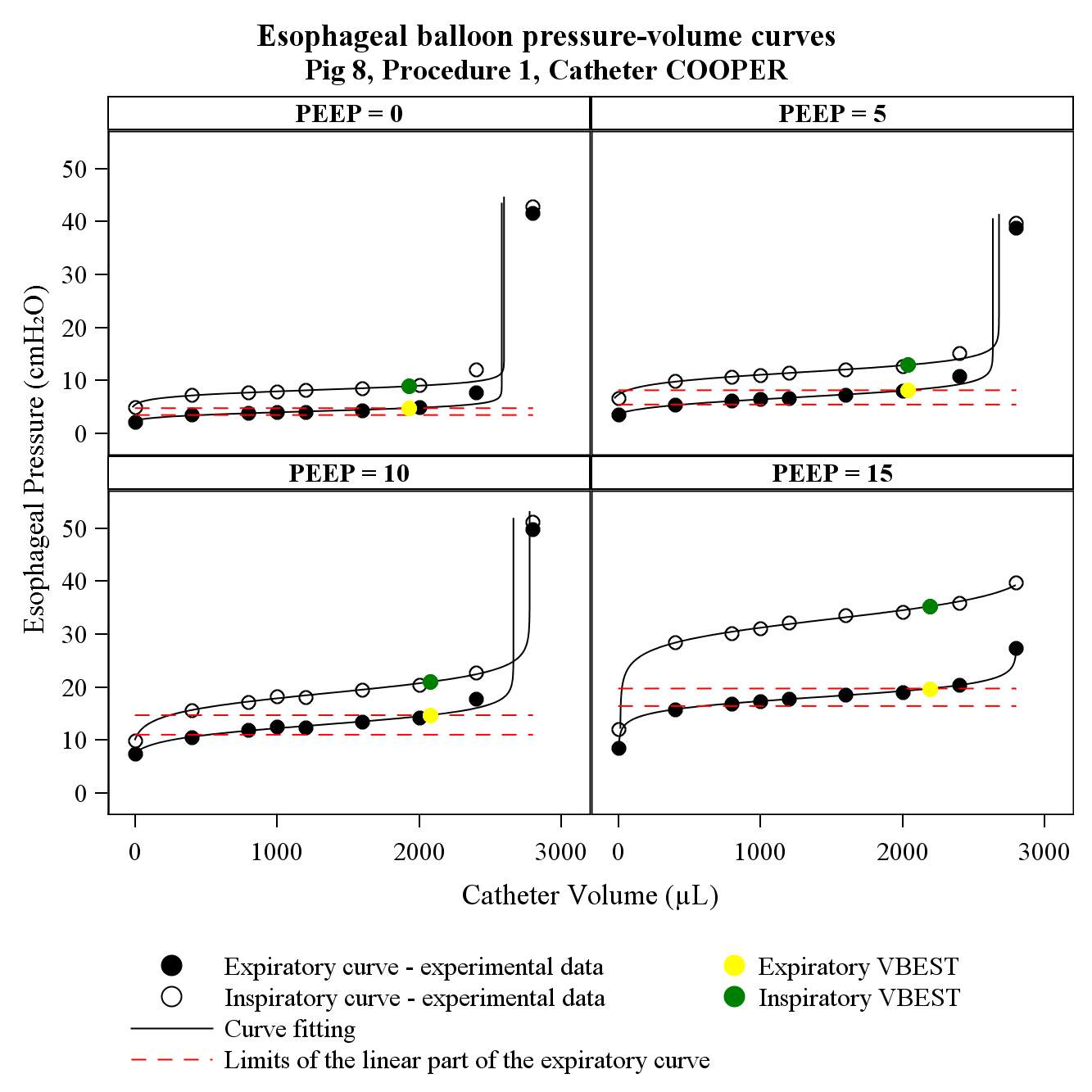


Limits of the quasi-linear part of the expiratory curve

**Figure E11:** *figure represents the esophageal balloon volume-pressure curves fittings at different PEEP levels (0, 5, 10, 15 cmH_2_O) measured during the application of manual incremental step inflation procedure with Nutrivent catheter for pig n°4. Dots represent experimental data points at end-inspiration (white), end-expiration (black), end-inspiratory V_BEST_ (yellow), end-inspiratory V_BEST_ (green). Continuous black lines represent fittings of the experimental data. Dashed red lines represent the limits of the quasi-linear part of the end-expiratory curve.*


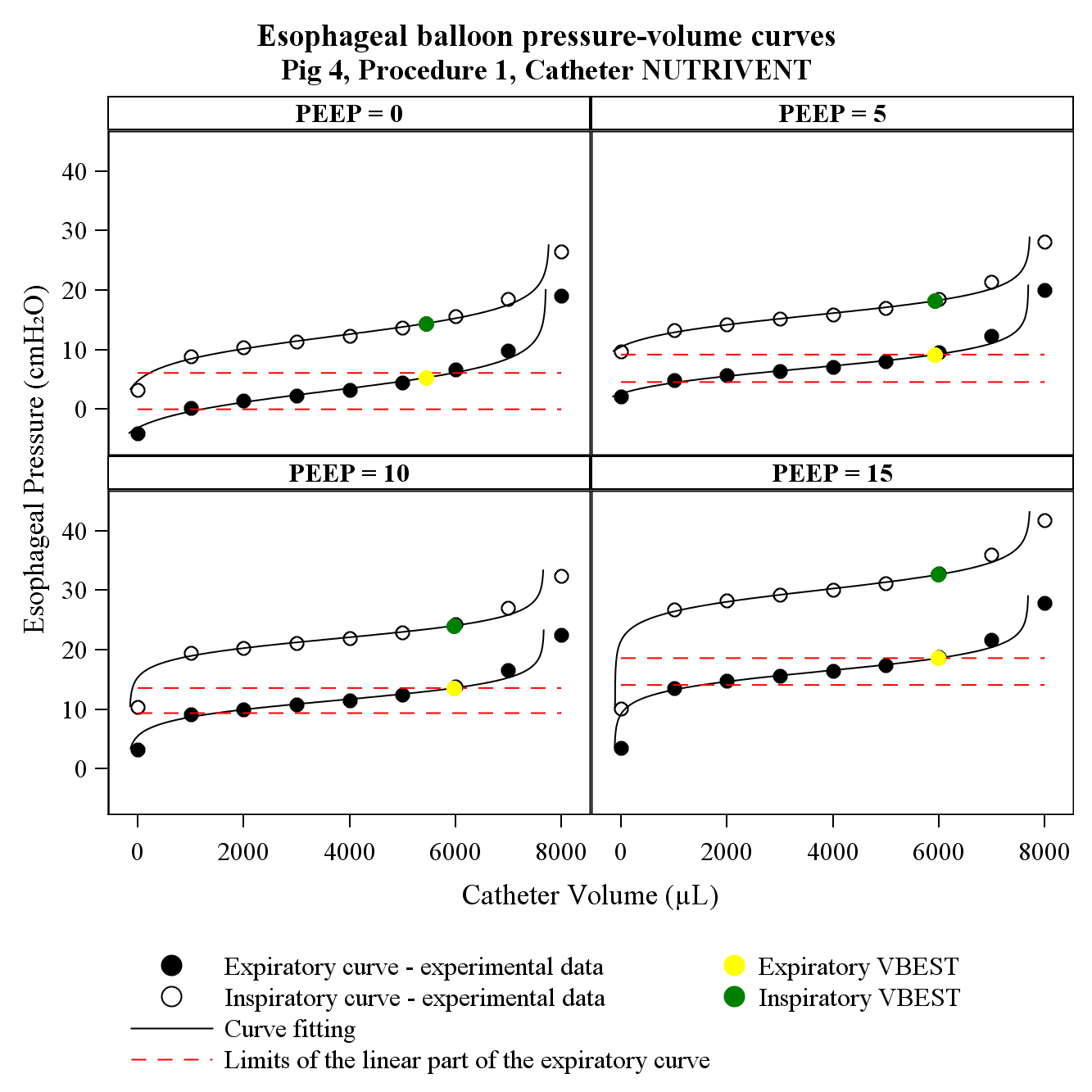


Limits of the quasi-linear part of the expiratory curve

**Figure E12:** *figure represents the esophageal balloon volume-pressure curves fittings at different PEEP levels (0, 5, 10, 15 cmH_2_O) measured during the application of manual incremental step inflation procedure with Nutrivent catheter for pig n°5. Dots represent experimental data points at end-inspiration (white), end-expiration (black), end-inspiratory V_BEST_ (yellow), end-inspiratory V_BEST_ (green). Continuous black lines represent fittings of the experimental data. Dashed red lines represent the limits of the quasi-linear part of the end-expiratory curve.*


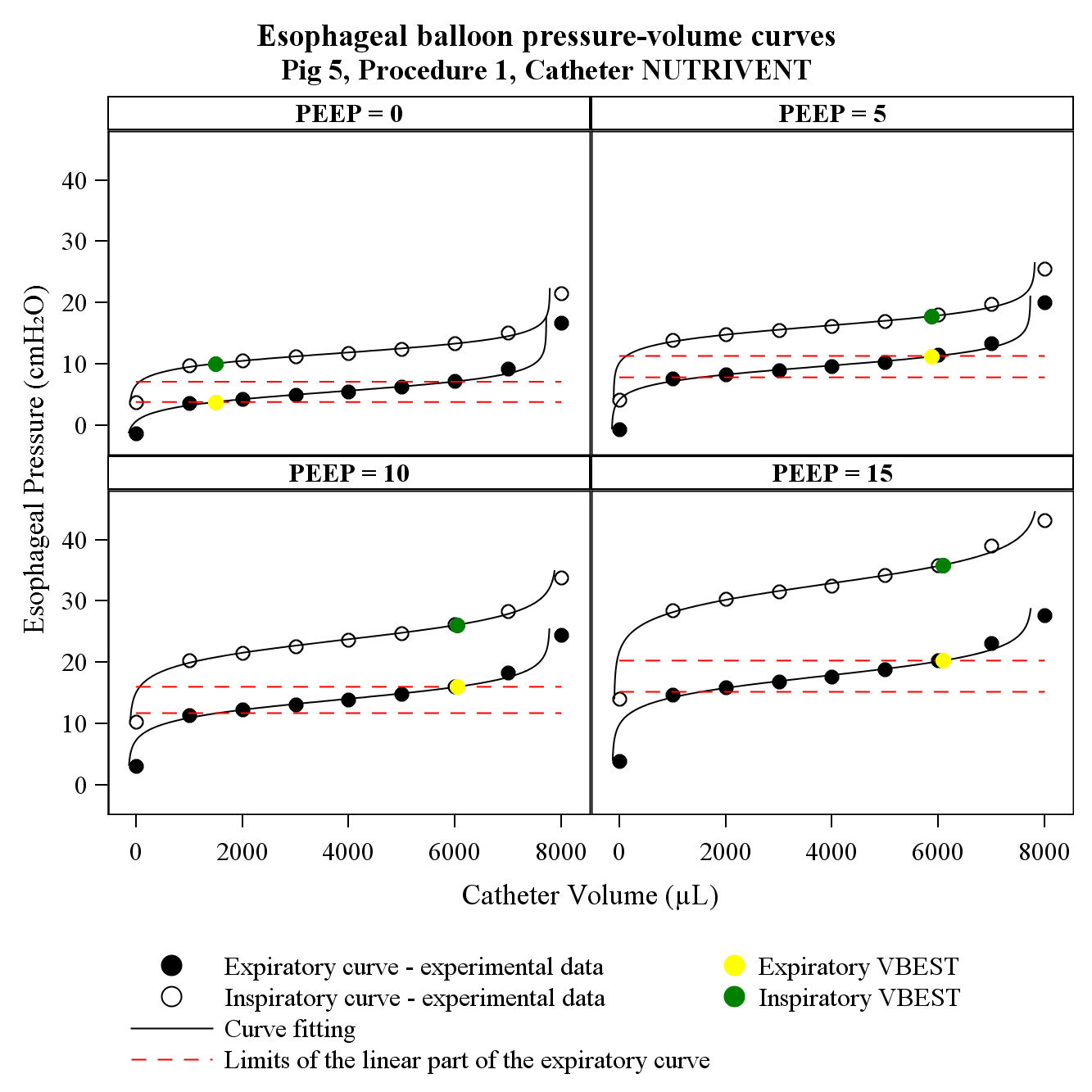


Limits of the quasi-linear part of the expiratory curve

**Figure E13:** *figure represents the esophageal balloon volume-pressure curves fittings at different PEEP levels (0, 5, 10, 15 cmH_2_O) measured during the application of manual incremental step inflation procedure with Nutrivent catheter for pig n°6. Dots represent experimental data points at end-inspiration (white), end-expiration (black), end-inspiratory V_BEST_ (yellow), end-inspiratory V_BEST_ (green). Continuous black lines represent fittings of the experimental data. Dashed red lines represent the limits of the quasi-linear part of the end-expiratory curve.*


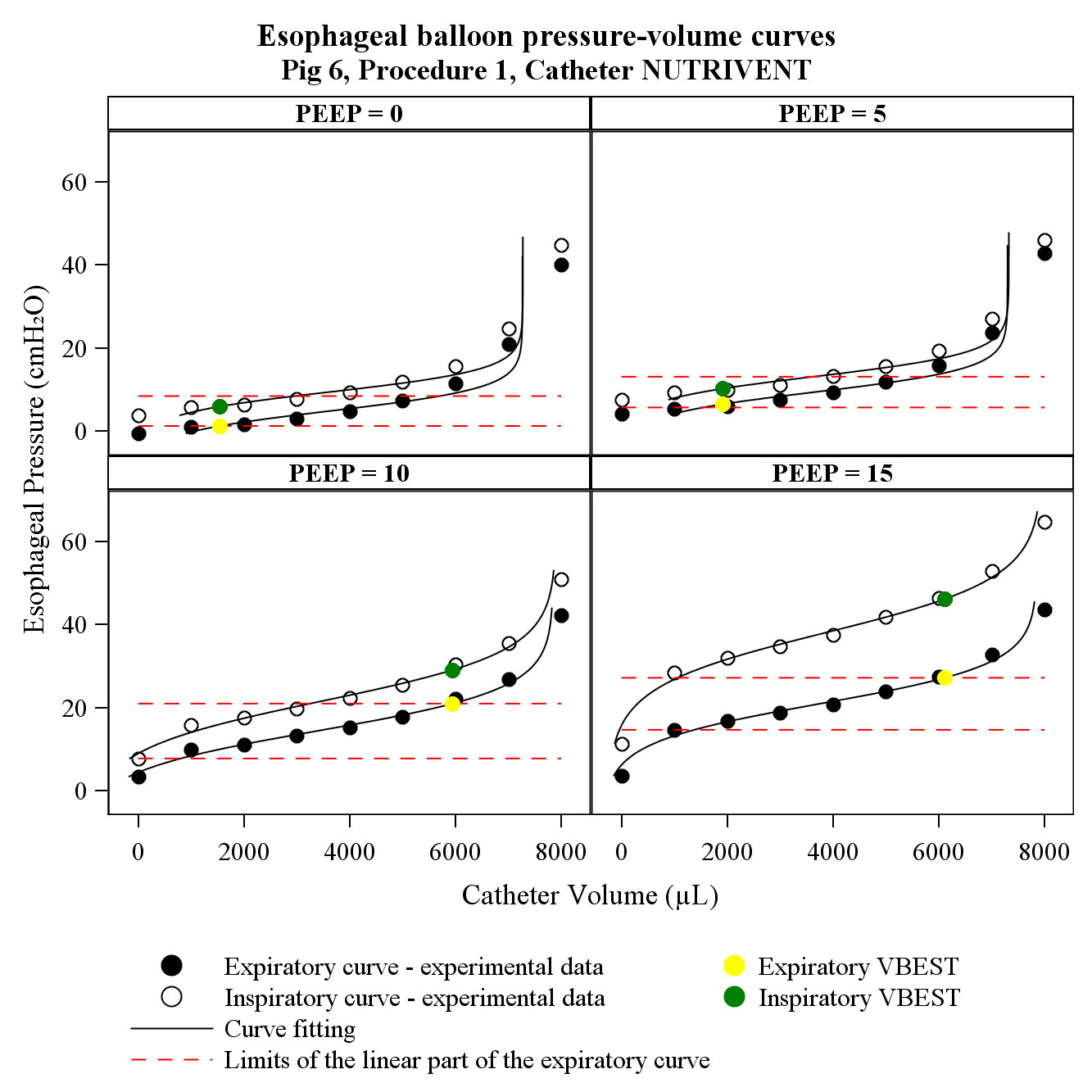


Limits of the quasi-linear part of the expiratory curve

**Figure E14:** *figure represents the esophageal balloon volume-pressure curves fittings at different PEEP levels (0, 5, 10, 15 cmH_2_O) measured during the application of manual incremental step inflation procedure with Nutrivent catheter for pig n°7. Dots represent experimental data points at end-inspiration (white), end-expiration (black), end-inspiratory V_BEST_ (yellow), end-inspiratory V_BEST_ (green). Continuous black lines represent fittings of the experimental data. Dashed red lines represent the limits of the quasi-linear part of the end-expiratory curve.*


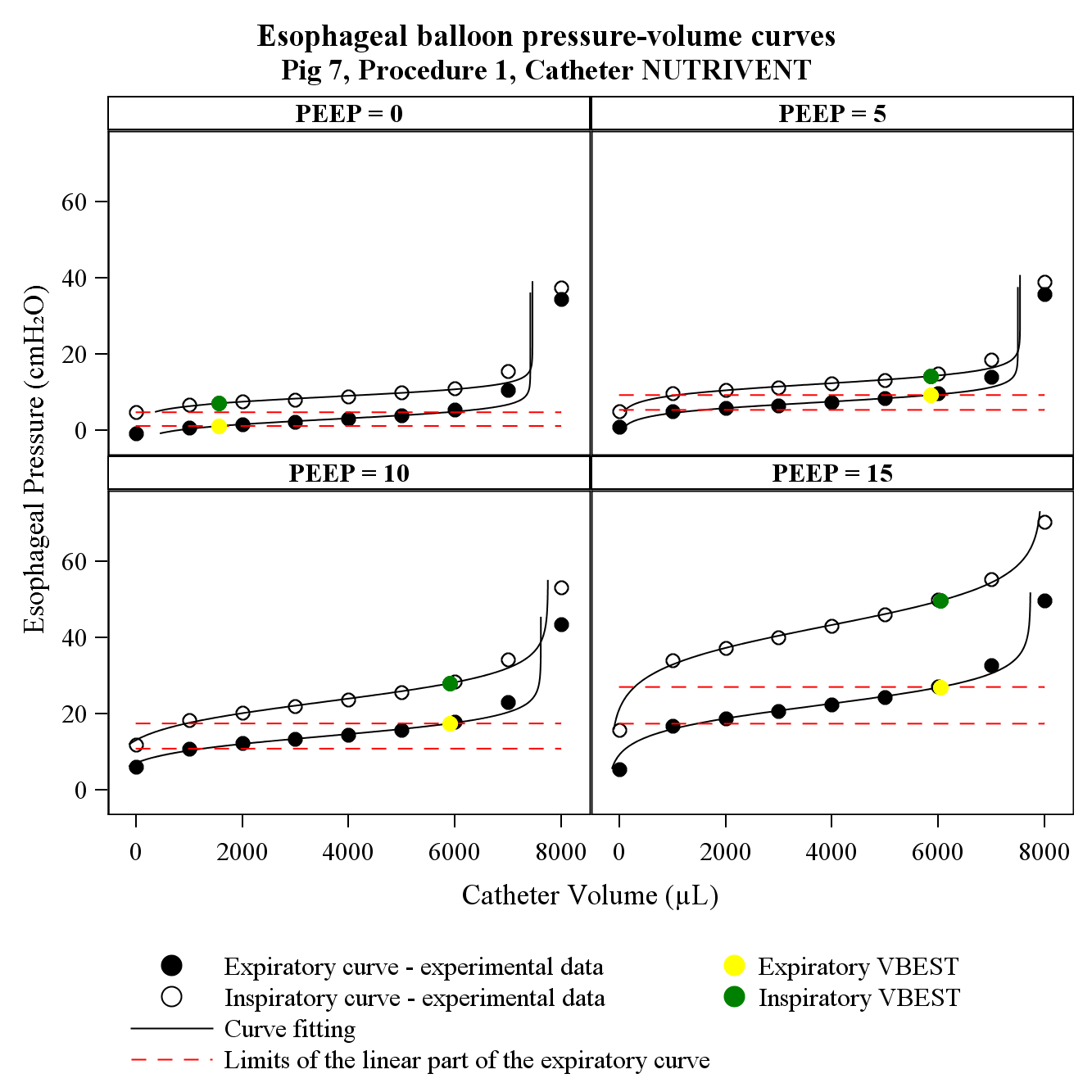


Limits of the quasi-linear part of the expiratory curve

**Figure E15:** *figure represents the esophageal balloon volume-pressure curves fittings at different PEEP levels (0, 5, 10, 15 cmH_2_O) measured during the application of manual incremental step inflation procedure with Nutrivent catheter for pig n°8. Dots represent experimental data points at end-inspiration (white), end-expiration (black), end-inspiratory V_BEST_ (yellow), end-inspiratory V_BEST_ (green). Continuous black lines represent fittings of the experimental data. Dashed red lines represent the limits of the quasi-linear part of the end-expiratory curve.*


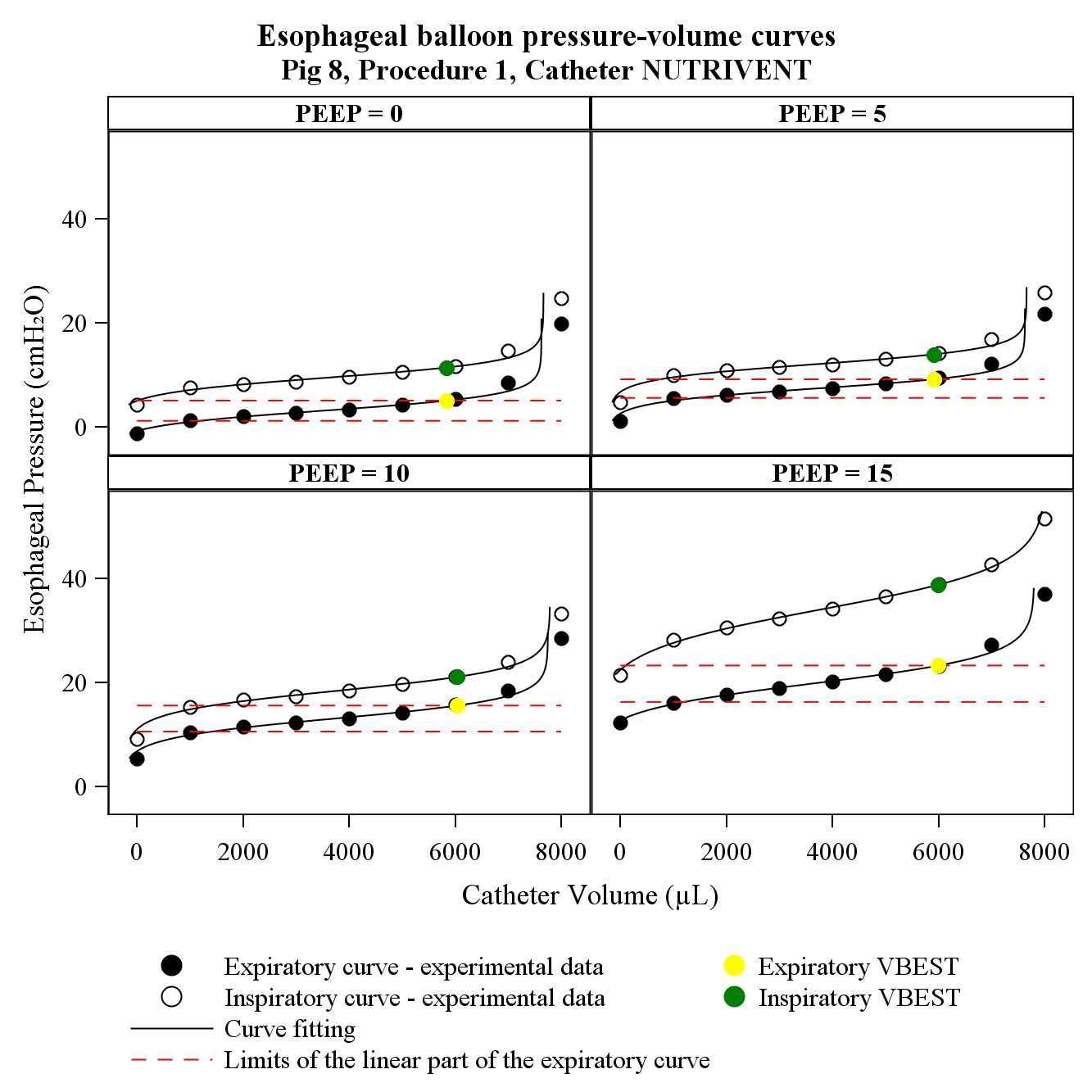


Limits of the quasi-linear part of the expiratory curve

### TABLE E1-E2. Parameters of end-expiratory sigmoidal curve, according to catheter.

*Mean ± standard deviation of* ***end-expiratory sigmoidal curve-fitting parameters*** *according to the catheter and the PEEP value tested.* ***a:*** *lower asymptote of balloon filling volume;* ***b:*** *distance from a to the upper asymptote of balloon filling volume;* ***c:*** *pressure of the true inflection point of the curve (where concavity changes direction);* ***d:*** *distance from* ***c*** *of the zone of high slope of the curve.*

**Table E1. Cooper catheter.**

|  | **PEEP (cmH_2_O)** | | | |
| --- | --- | --- | --- | --- |
|  | **0** | **5** | **10** | **15** |
| **a**  **(μL)** | -253.3 | -57.8 | -53.9 | -29.3 |
|  | ± | ± | ± | ± |
|  | 251.8 | 54.3 | 21.3 | 21.9 |
| **b**  **(μL)** | 2974.2 | 2735.5 | 2756.1 | 2822.1 |
|  | ± | ± | ± | ± |
|  | 279.2 | 48.4 | 37.1 | 118.8 |
| **d (cmH_2_O)** | 4.7 | 8.7 | 14.3 | 19.0 |
|  | ± | ± | ± | ± |
|  | 2.7 | 2.5 | 2.6 | 1.2 |
| **c (cmH_2_O)** | 1.5 | 1.3 | 1.7 | 1.8 |
|  | ± | ± | ± | ± |
|  | 0.8 | 0.3 | 0.5 | 0.6 |
| **R²** | 0.990 | 0.990 | 0.990 | 0.990 |
|  | ± | ± | ± | ± |
|  | 0.010 | 0.000 | 0.000 | 0.010 |

**Table E2. Nutrivent catheter.**

|  | **PEEP (cmH_2_O)** | | | |
| --- | --- | --- | --- | --- |
|  | **0** | **0** | **0** | **0** |
| **a**  **(μL)** | -256.7 | -210.1 | -428.2 | -277.4 |
|  | ± | ± | ± | ± |
|  | 280.5 | 278.4 | 382.2 | 186.1 |
| **b**  **(μL)** | 7805.6 | 7781.8 | 8163.0 | 8048.7 |
|  | ± | ± | ± | ± |
|  | 446.2 | 410.6 | 434.8 | 232.9 |
| **c**  **(cmH_2_O)** | 3.9 | 8.1 | 13.4 | 19.4 |
|  | ± | ± | ± | ± |
|  | 1.2 | 1.3 | 1.2 | 2.4 |
| **d**  **(cmH_2_O)** | 1.9 | 1.8 | 2.6 | 3.0 |
|  | ± | ± | ± | ± |
|  | 0.7 | 0.6 | 1.4 | 1.3 |
| **R²** | 0.980 | 0.980 | 0.990 | 0.990 |
|  | ± | ± | ± | ± |
|  | 0.020 | 0.020 | 0.000 | 0.000 |

### TABLE E3-E4. Parameters of end-inspiratory sigmoidal curve, according to catheter.

*Mean ± standard deviation of* ***end-inspiratory sigmoidal curve-fitting parameters*** *according to the catheter, the procedure and the PEEP value tested.* ***a:*** *lower asymptote of balloon filling volume;* ***b:*** *distance from a to the upper asymptote of balloon filling volume;* ***c:*** *pressure of the true inflection point of the curve;* ***d:*** *distance from* ***c*** *of the zone of high slope of the curve.*

**Table E3. Cooper catheter.**

|  | **PEEP (cmH_2_O)** | | | |
| --- | --- | --- | --- | --- |
|  | **0** | **5** | **10** | **15** |
| **a**  **(μL)** | -118.6 | -31.1 | -31.2 | 3.2 |
|  | ± | ± | ± | ± |
|  | 135.4 | 41.7 | 12.2 | 31.1 |
| **b**  **(μL)** | 2823.9 | 2752.2 | 2785.0 | 2959.1 |
|  | ± | ± | ± | ± |
|  | 179.9 | 62.8 | 44.8 | 147.3 |
| **c (cmH_2_O)** | 9.3 | 13.2 | 21.9 | 35.3 |
|  | ± | ± | ± | ± |
|  | 2.5 | 3.5 | 4.5 | 4.9 |
| **d (cmH_2_O)** | 1.3 | 1.5 | 2.1 | 3.1 |
|  | ± | ± | ± | ± |
|  | 0.5 | 0.4 | 0.8 | 1.2 |
| **R²** | 0.990 | 0.990 | 0.990 | 0.990 |
|  | ± | ± | ± | ± |
|  | 0.010 | 0.000 | 0.000 | 0.010 |

**Table E4. Nutrivent catheter.**

|  | **PEEP (cmH_2_O)** | | | |
| --- | --- | --- | --- | --- |
|  | **0** | **5** | **10** | **15** |
| **a**  **(μL)** | -174.0 | -158.4 | -394.4 | -224.6 |
|  | ± | ± | ± | ± |
|  | 196.2 | 198.8 | 351.8 | 158.8 |
| **b**  **(μL)** | 7766.2 | 7773.0 | 8198.1 | 8138.5 |
|  | ± | ± | ± | ± |
|  | 378.0 | 344.8 | 402.2 | 256.8 |
| **c**  **(cmH_2_O)** | 10.3 | 13.9 | 21.7 | 35.5 |
|  | ± | ± | ± | ± |
|  | 1.6 | 2.0 | 2.0 | 5.0 |
| **d**  **(cmH_2_O)** | 1.8 | 1.9 | 3.1 | 4.2 |
|  | ± | ± | ± | ± |
|  | 0.6 | 0.6 | 1.8 | 2.0 |
| **R²** | 0.990 | 0.990 | 0.990 | 1.000 |
|  | ± | ± | ± | ± |
|  | 0.010 | 0.010 | 0.000 | 0.000 |

### TABLE E5-E6. Parameters estimated during the CORRECTION, according to catheter.

*Mean ± standard deviation of* ***parameters estimated during the correction*** *(see Supplementary Materials and Methods)* ***and of intrathoracic pressures*** *according to the catheter and the PEEP value tested.*

**Table E5. Cooper catheter.**

|  | **PEEP (cmH₂O)** | | | |
| --- | --- | --- | --- | --- |
|  | **0** | **5** | **10** | **15** |
| **V_MIN_**  **(µL)** | 375.3 | 520.2 | 528.5 | 567.1 |
|  | ± | ± | ± | ± |
|  | 208.3 | 52.9 | 28.7 | 27.9 |
| **V_MAX_**  **(µL)** | 2063.2 | 2083.8 | 2109.3 | 2183.0 |
|  | ± | ± | ± | ± |
|  | 170.0 | 64.7 | 47.5 | 91.0 |
| **P_MIN_ (cmH₂O)** | 2.80 | 6.96 | 12.14 | 16.61 |
|  | ± | ± | ± | ± |
|  | 2.83 | 2.35 | 2.10 | 0.82 |
| **P_MAX_ (cmH₂O)** | 6.60 | 10.36 | 16.48 | 21.31 |
|  | ± | ± | ± | ± |
|  | 3.06 | 2.70 | 3.19 | 1.77 |
| **V_BEST_**  **(µL)** | 1341.6 | 2083.8 | 2109.3 | 2183.0 |
|  | ± | ± | ± | ± |
|  | 780.3 | 64.7 | 47.5 | 91.0 |
| **P_EE_ _BEST_ (cmH₂O)** | 4.46 | 10.36 | 16.48 | 21.31 |
|  | ± | ± | ± | ± |
|  | 2.16 | 2.70 | 3.19 | 1.77 |
| **P_EI BEST_ (cmH₂O)** | 9.01 | 14.91 | 24.46 | 38.43 |
|  | ± | ± | ± | ± |
|  | 1.82 | 3.77 | 5.35 | 5.36 |
| **P_BEST_ _SWINGS_ (cmH₂O)** | 4.55 | 4.56 | 7.98 | 17.12 |
|  | ± | ± | ± | ± |
|  | 0.61 | 1.24 | 2.17 | 4.43 |
| **E_ES_ (cmH₂O/mL)** | 2.17 | 2.17 | 2.74 | 2.93 |
|  | ± | ± | ± | ± |
|  | 1.01 | 0.44 | 0.84 | 0.98 |
| **P_EW_ (cmH₂O)** | 1.64 | 3.40 | 4.34 | 4.70 |
|  | ± | ± | ± | ± |
|  | 1.18 | 0.72 | 1.36 | 1.50 |
| **P_EE BEST_ CORR.**  **(cmH₂O)** | 2.82 | 6.96 | 12.14 | 16.61 |
|  | ± | ± | ± | ± |
|  | 2.79 | 2.35 | 2.10 | 0.82 |
| **P_EI BEST_ CORR. (cmH₂O)** | 7.37 | 11.51 | 20.12 | 33.73 |
|  | ± | ± | ± | ± |
|  | 2.57 | 3.41 | 4.19 | 4.93 |
| **P_EE_ _IT_  (cmH₂O)** | 1.88 | 6.24 | 10.55 | 14.57 |
|  | ± | ± | ± | ± |
|  | 2.20 | 2.37 | 2.54 | 2.64 |
| **P_EI_ _IT_ (cmH₂O)** | 7.11 | 10.52 | 16.42 | 27.83 |
|  | ± | ± | ± | ± |
|  | 2.08 | 2.97 | 3.77 | 4.11 |
| **P_IT_ _SWINGS_ (cmH₂O)** | 5.22 | 4.28 | 5.87 | 13.25 |
|  | ± | ± | ± | ± |
|  | 0.79 | 0.88 | 1.26 | 2.00 |

**Table E6. Nutrivent catheter.**

|  | **PEEP (cmH₂O)** | | | |
| --- | --- | --- | --- | --- |
|  | **0** | **5** | **10** | **15** |
| **V_MIN_**  **(µL)** | 1392.8 | 1434.3 | 1296.8 | 1423.4 |
|  | ± | ± | ± | ± |
|  | 192.9 | 199.0 | 292.3 | 138.2 |
| **V_MAX_**  **(µL)** | 5859.0 | 5883.2 | 5978.8 | 6045.6 |
|  | ± | ± | ± | ± |
|  | 108.3 | 85.7 | 55.7 | 56.0 |
| **P_MIN_ (cmH₂O)** | 1.42 | 5.76 | 10.01 | 15.49 |
|  | ± | ± | ± | ± |
|  | 1.39 | 1.20 | 1.51 | 1.30 |
| **P_MAX_ (cmH₂O)** | 6.24 | 10.36 | 16.67 | 23.23 |
|  | ± | ± | ± | ± |
|  | 1.53 | 1.76 | 2.75 | 3.86 |
| **V_BEST_**  **(µL)** | 3175.2 | 5096.0 | 5978.8 | 6045.6 |
|  | ± | ± | ± | ± |
|  | 2254.9 | 1777.7 | 55.7 | 56.0 |
| **P_EE BEST_ (cmH₂O)** | 3.26 | 9.02 | 16.67 | 23.23 |
|  | ± | ± | ± | ± |
|  | 2.04 | 1.67 | 2.75 | 3.86 |
| **P_EI BEST_ (cmH₂O)** | 9.74 | 14.84 | 25.63 | 40.58 |
|  | ± | ± | ± | ± |
|  | 3.43 | 3.26 | 3.18 | 7.08 |
| **P_BEST SWINGS_ (cmH₂O)** | 6.48 | 5.82 | 8.95 | 17.36 |
|  | ± | ± | ± | ± |
|  | 1.62 | 2.06 | 2.17 | 3.47 |
| **E_ES_ (cmH₂O/mL)** | 1.09 | 1.04 | 1.39 | 1.67 |
|  | ± | ± | ± | ± |
|  | 0.42 | 0.41 | 0.69 | 0.69 |
| **P_EW_ (cmH₂O)** | 1.87 | 3.24 | 6.66 | 7.74 |
|  | ± | ± | ± | ± |
|  | 2.61 | 1.51 | 3.78 | 3.32 |
| **P_EE BEST_ CORR. (cmH₂O)** | 1.40 | 5.79 | 10.01 | 15.49 |
|  | ± | ± | ± | ± |
|  | 1.43 | 1.19 | 1.51 | 1.30 |
| **P_EI BEST_ CORR. (cmH₂O)** | 7.87 | 11.61 | 18.97 | 32.84 |
|  | ± | ± | ± | ± |
|  | 1.63 | 2.18 | 2.84 | 4.46 |
| **P_EE IT_ (cmH₂O)** | 1.12 | 5.60 | 10.12 | 14.59 |
|  | ± | ± | ± | ± |
|  | 1.78 | 1.69 | 1.46 | 1.07 |
| **P_EI IT_ (cmH₂O)** | 8.22 | 11.84 | 18.32 | 28.91 |
|  | ± | ± | ± | ± |
|  | 2.09 | 2.79 | 3.24 | 1.97 |
| **P_IT_ _SWINGS_ (cmH₂O)** | 7.10 | 6.24 | 8.20 | 14.32 |
|  | ± | ± | ± | ± |
|  | 1.46 | 1.93 | 2.32 | 1.20 |

**Abbreviations of table E5-E6.**

**V_MIN_:** the lower limit of *quasi-*linear portion of the end-expiratory VP curve, it represents the smallest filling volume to pressurize the catheter at the same pressure surrounding the esophageal balloon;

**V_MAX:_** the upper limit of *quasi-*linear portion of the end-expiratory VP curve, it represents the larger filling volume that does not induce overstretch of the balloon wall;

**P_MIN_:** pressure on the end-expiratory VP curve corresponding to V_MIN_;

**P_MAX_:** pressure on the end-expiratory VP curve corresponding to V_MAX_;

**V_BEST_:** the optimal catheter filling volume;

**P_EE BEST_:** pressure on the end-expiratory VP curve corresponding to V_BEST_;

**P_EI BEST_:** pressure on the end-inspiratory VP curve corresponding to V_BEST_;

**P_BEST SWINGS_:** the difference between P_EI BEST_ and P_EE BEST;_

**E_ES_:** elastance of the esophagus, computed according to eq. 3 (see supplementary materials and methods);

**P_EW_:** pressure generated by the esophagus wall calculated according to eq. 2;

**P_EE BEST_ CORR:** corrected end-expiratory esophageal pressures obtained at V_BEST_ (P_EE BEST_ - P_EW);_

**P_EI BEST_ CORR:** corrected end-inspiratory esophageal pressures obtained at V_BEST_ (P_EE BEST_ - P_EW);_

**P_EE IT_:** intrathoracic end-expiratory pressure;

**P_EI IT_:** intrathoracic end-inspiratory pressure;

**P_IT_ SWINGS:** P_EI IT_ - P_EE IT._

### TABLE E7-E16. LINEAR REGRESSIONS RESULTS

***Table E7-E8. Parameters obtained by linear regression models*** *between end-expiratory intrathoracic pressure and* ***uncorrected*** ***end-expiratory esophageal pressure*** *according to the catheter tested. Regressions were performed for the overall data, for different catheters and procedures with overall PEEP values applied and at each individual PEEP value (0, 5, 10 and 15 cmH_2_O). Equation: f = y0+a*x. Parm. indicates parameters, CI indicates confidence interval, Rsqr indicates R*^2^*, Adj indicates adjusted.*

| **catheter** | **procedure** | **PEEP** | **Coefficient** | **Parm** | **Std. Error** | **t** | ***P*** | **95% CI** | | **Rsqr** | **Adj Rsqr** | ***P*** |
| --- | --- | --- | --- | --- | --- | --- | --- | --- | --- | --- | --- | --- |
| **ALL** | **ALL** | **ALL** | **y0** | 2.39 | 0.74 | 3.22 | 0.002 | 0.92 | 3.86 | 0.73 | 0.73 | <0.001 |
|  |  |  | **a** | 1.38 | 0.08 | 17.77 | <0.001 | 1.22 | 1.53 |  |  |  |
| **COOPER** | **MANUAL INCREMENTAL STEP INFLATION** | **ALL** | **y0** | 3.11 | 1.05 | 2.96 | 0.008 | 0.91 | 5.32 | 0.88 | 0.87 | <0.001 |
|  |  |  | **a** | 1.21 | 0.11 | 11.28 | <0.001 | 0.98 | 1.43 |  |  |  |
|  |  | **0** | **y0** | 3.02 | 0.96 | 3.13 | 0.052 | -0.05 | 6.09 | 0.61 | 0.48 | 0.120 |
|  |  |  | **a** | 0.76 | 0.35 | 2.16 | 0.120 | -0.36 | 1.89 |  |  |  |
|  |  | **5** | **y0** | 3.90 | 1.81 | 2.16 | 0.120 | -1.86 | 9.66 | 0.83 | 0.77 | 0.033 |
|  |  |  | **a** | 1.03 | 0.27 | 3.77 | 0.033 | 0.16 | 1.91 |  |  |  |
|  |  | **10** | **y0** | 6.07 | 4.85 | 1.25 | 0.299 | -9.35 | 21.49 | 0.62 | 0.49 | 0.115 |
|  |  |  | **a** | 0.99 | 0.45 | 2.20 | 0.115 | -0.44 | 2.42 |  |  |  |
|  |  | **15** | **y0** | 24.93 | 5.31 | 4.70 | 0.018 | 8.03 | 41.83 | 0.14 | -0.15 | 0.540 |
|  |  |  | **a** | -0.25 | 0.36 | -0.69 | 0.540 | -1.39 | 0.90 |  |  |  |

| **catheter** | **procedure** | **PEEP** | **Coefficient** | **Parm** | **Std. Error** | **t** | ***P*** | **95% CI** | | **Rsqr** | **Adj Rsqr** | ***P*** |
| --- | --- | --- | --- | --- | --- | --- | --- | --- | --- | --- | --- | --- |
| **ALL** | **ALL** | **ALL** | **y0** | 2.39 | 0.74 | 3.22 | 0.002 | 0.92 | 3.86 | 0.73 | 0.73 | <0.001 |
|  |  |  | **a** | 1.38 | 0.08 | 17.77 | <0.001 | 1.22 | 1.53 |  |  |  |
| **NUTRIVENT** | **MANUAL INCREMENTAL STEP INFLATION** | **ALL** | **y0** | 2.09 | 1.37 | 1.52 | 0.147 | -0.80 | 4.97 | 0.84 | 0.83 | <0.001 |
|  |  |  | **a** | 1.39 | 0.15 | 9.56 | <0.001 | 1.09 | 1.70 |  |  |  |
|  |  | **0** | **y0** | 3.22 | 1.29 | 2.50 | 0.088 | -0.88 | 7.32 | 0.00 | -0.33 | 0.958 |
|  |  |  | **a** | 0.04 | 0.66 | 0.06 | 0.958 | -2.07 | 2.15 |  |  |  |
|  |  | **5** | **y0** | 6.07 | 2.81 | 2.16 | 0.120 | -2.88 | 15.03 | 0.28 | 0.04 | 0.357 |
|  |  |  | **a** | 0.53 | 0.49 | 1.09 | 0.357 | -1.02 | 2.07 |  |  |  |
|  |  | **10** | **y0** | 17.65 | 11.11 | 1.59 | 0.210 | -17.71 | 53.01 | 0.00 | -0.33 | 0.935 |
|  |  |  | **a** | -0.10 | 1.09 | -0.09 | 0.935 | -3.56 | 3.37 |  |  |  |
|  |  | **15** | **y0** | 51.32 | 25.79 | 1.99 | 0.141 | -30.75 | 133.39 | 0.28 | 0.05 | 0.355 |
|  |  |  | **a** | -1.93 | 1.76 | -1.09 | 0.355 | -7.54 | 3.69 |  |  |  |

***Table E9-E10. Parameters obtained by linear regression models*** *between end-expiratory intrathoracic pressure and* ***corrected*** ***end-expiratory esophageal pressure*** *according to the catheter tested (esophageal balloon inflated at V_BEST_). Regressions were performed for the overall data, for different catheters with overall PEEP values applied and at each individual PEEP value (0, 5, 10 and 15 cmH_2_O). Equation: f = y0+a*x. Parm. indicates parameters, CI indicates confidence interval, Rsqr indicates R*^2^*, Adj indicates adjusted.*

| **catheter** | **procedure** | **PEEP** | **Coefficient** | **Parm** | **Std. Error** | **t** | ***P*** | **95% CI** | | **Rsqr** | **Adj Rsqr** | ***P*** |
| --- | --- | --- | --- | --- | --- | --- | --- | --- | --- | --- | --- | --- |
| **ALL** | **ALL** | **ALL** | **y0** | 0.96 | 0.41 | 2.32 | 0.022 | 0.14 | 1.78 | 0.83 | 0.83 | <0.001 |
|  |  |  | **a** | 1.04 | 0.04 | 24.01 | <0.001 | 0.95 | 1.12 |  |  |  |
| **COOPER** | **MANUAL INCREMENTAL STEP INFLATION** | **ALL** | **y0** | 1.26 | 0.79 | 1.59 | 0.129 | -0.40 | 2.92 | 0.90 | 0.89 | <0.001 |
|  |  |  | **a** | 1.01 | 0.08 | 12.49 | <0.001 | 0.84 | 1.18 |  |  |  |
|  |  | **0** | **y0** | 0.75 | 0.99 | 0.76 | 0.504 | -2.41 | 3.91 | 0.75 | 0.67 | 0.057 |
|  |  |  | **a** | 1.10 | 0.36 | 3.01 | 0.057 | -0.06 | 2.26 |  |  |  |
|  |  | **5** | **y0** | 0.87 | 0.69 | 1.25 | 0.299 | -1.34 | 3.08 | 0.97 | 0.95 | 0.003 |
|  |  |  | **a** | 0.98 | 0.11 | 9.26 | 0.003 | 0.64 | 1.31 |  |  |  |
|  |  | **10** | **y0** | 4.02 | 1.87 | 2.15 | 0.120 | -1.92 | 9.95 | 0.87 | 0.83 | 0.021 |
|  |  |  | **a** | 0.77 | 0.17 | 4.46 | 0.021 | 0.22 | 1.32 |  |  |  |
|  |  | **15** | **y0** | 20.58 | 1.26 | 16.31 | 0.001 | 16.57 | 24.60 | 0.77 | 0.70 | 0.050 |
|  |  |  | **a** | -0.27 | 0.09 | -3.19 | 0.050 | -0.54 | 0.00 |  |  |  |

| **catheter** | **procedure** | **PEEP** | **Coefficient** | **Parm** | **Std. Error** | **t** | ***P*** | **95% CI** | | **Rsqr** | **Adj Rsqr** | ***P*** |
| --- | --- | --- | --- | --- | --- | --- | --- | --- | --- | --- | --- | --- |
| **ALL** | **ALL** | **ALL** | **y0** | 0.96 | 0.41 | 2.32 | 0.022 | 0.14 | 1.78 | 0.83 | 0.83 | <0.001 |
|  |  |  | **a** | 1.04 | 0.04 | 24.01 | <0.001 | 0.95 | 1.12 |  |  |  |
| **NUTRIVENT** | **MANUAL INCREMENTAL STEP INFLATION** | **ALL** | **y0** | 0.30 | 0.51 | 0.58 | 0.567 | -0.77 | 1.37 | 0.95 | 0.95 | <0.001 |
|  |  |  | **a** | 1.00 | 0.05 | 18.50 | <0.001 | 0.89 | 1.12 |  |  |  |
|  |  | **0** | **y0** | 0.55 | 0.31 | 1.79 | 0.172 | -0.43 | 1.53 | 0.88 | 0.84 | 0.017 |
|  |  |  | **a** | 0.75 | 0.16 | 4.77 | 0.017 | 0.25 | 1.26 |  |  |  |
|  |  | **5** | **y0** | 2.01 | 0.72 | 2.81 | 0.067 | -0.27 | 4.29 | 0.91 | 0.88 | 0.012 |
|  |  |  | **a** | 0.67 | 0.12 | 5.46 | 0.012 | 0.28 | 1.07 |  |  |  |
|  |  | **10** | **y0** | 4.83 | 5.33 | 0.91 | 0.432 | -12.14 | 21.80 | 0.24 | -0.01 | 0.399 |
|  |  |  | **a** | 0.51 | 0.52 | 0.98 | 0.399 | -1.15 | 2.18 |  |  |  |
|  |  | **15** | **y0** | 18.96 | 10.08 | 1.88 | 0.157 | -13.12 | 51.04 | 0.04 | -0.28 | 0.753 |
|  |  |  | **a** | -0.24 | 0.69 | -0.35 | 0.753 | -2.43 | 1.96 |  |  |  |

***Table E11-E12. Parameters obtained by linear regression models*** *between end-inspiratory intrathoracic pressure and* ***uncorrected*** ***end-inpiratory esophageal*** *according to the catheter tested. Regressions were performed for the overall data, for different catheters with overall PEEP values applied and at each individual PEEP value (0, 5, 10 and 15 cmH_2_O). Equation: f = y0+a*x. Parm. indicates parameters, CI indicates confidence interval, Rsqr indicates R*^2^*, Adj indicates adjusted.*

| **catheter** | **procedure** | **PEEP** | **Coefficient** | **Parm** | **Std. Error** | **t** | ***P*** | **95% CI** | | **Rsqr** | **Adj Rsqr** | ***P*** |
| --- | --- | --- | --- | --- | --- | --- | --- | --- | --- | --- | --- | --- |
| **ALL** | **ALL** | **ALL** | **y0** | 0.24 | 1.11 | 0.22 | 0.830 | -1.97 | 2.45 | 0.81 | 0.80 | <0.001 |
|  |  |  | **a** | 1.34 | 0.06 | 21.99 | <0.001 | 1.22 | 1.46 |  |  |  |
| **COOPER** | **MANUAL INCREMENTAL STEP INFLATION** | **ALL** | **y0** | 1.90 | 2.34 | 0.81 | 0.427 | -3.02 | 6.82 | 0.84 | 0.83 | <0.001 |
|  |  |  | **a** | 1.28 | 0.13 | 9.63 | <0.001 | 1.00 | 1.56 |  |  |  |
|  |  | **0** | **y0** | 3.72 | 1.93 | 1.92 | 0.150 | -2.44 | 9.87 | 0.73 | 0.64 | 0.066 |
|  |  |  | **a** | 0.74 | 0.26 | 2.83 | 0.066 | -0.09 | 1.58 |  |  |  |
|  |  | **5** | **y0** | 2.52 | 3.00 | 0.84 | 0.462 | -7.02 | 12.06 | 0.86 | 0.81 | 0.024 |
|  |  |  | **a** | 1.18 | 0.28 | 4.27 | 0.024 | 0.30 | 2.06 |  |  |  |
|  |  | **10** | **y0** | 5.28 | 7.79 | 0.68 | 0.547 | -19.53 | 30.08 | 0.68 | 0.57 | 0.087 |
|  |  |  | **a** | 1.17 | 0.47 | 2.51 | 0.087 | -0.31 | 2.65 |  |  |  |
|  |  | **15** | **y0** | 59.86 | 17.06 | 3.51 | 0.039 | 5.55 | 114.16 | 0.35 | 0.13 | 0.295 |
|  |  |  | **a** | -0.77 | 0.61 | -1.27 | 0.295 | -2.71 | 1.17 |  |  |  |

| **catheter** | **procedure** | **PEEP** | **Coefficient** | **Parm** | **Std. Error** | **t** | ***P*** | **95% CI** | | **Rsqr** | **Adj Rsqr** | ***P*** |
| --- | --- | --- | --- | --- | --- | --- | --- | --- | --- | --- | --- | --- |
| **ALL** | **ALL** | **ALL** | **y0** | 0.24 | 1.11 | 0.22 | 0.830 | -1.97 | 2.45 | 0.81 | 0.80 | <0.001 |
|  |  |  | **a** | 1.34 | 0.06 | 21.99 | <0.001 | 1.22 | 1.46 |  |  |  |
| **NUTRIVENT** | **MANUAL INCREMENTAL STEP INFLATION** | **ALL** | **y0** | -1.08 | 2.52 | -0.43 | 0.672 | -6.37 | 4.20 | 0.86 | 0.85 | <0.001 |
|  |  |  | **a** | 1.41 | 0.13 | 10.52 | <0.001 | 1.13 | 1.70 |  |  |  |
|  |  | **0** | **y0** | 1.94 | 6.50 | 0.30 | 0.785 | -18.75 | 22.64 | 0.34 | 0.11 | 0.306 |
|  |  |  | **a** | 0.95 | 0.77 | 1.23 | 0.306 | -1.51 | 3.40 |  |  |  |
|  |  | **5** | **y0** | 3.36 | 4.52 | 0.74 | 0.511 | -11.03 | 17.75 | 0.69 | 0.59 | 0.081 |
|  |  |  | **a** | 0.97 | 0.37 | 2.60 | 0.081 | -0.22 | 2.16 |  |  |  |
|  |  | **10** | **y0** | 24.75 | 10.50 | 2.36 | 0.100 | -8.67 | 58.18 | 0.00 | -0.33 | 0.938 |
|  |  |  | **a** | 0.05 | 0.57 | 0.08 | 0.938 | -1.76 | 1.85 |  |  |  |
|  |  | **15** | **y0** | 53.49 | 59.83 | 0.89 | 0.437 | -136.91 | 243.90 | 0.02 | -0.31 | 0.843 |
|  |  |  | **a** | -0.45 | 2.07 | -0.22 | 0.843 | -7.02 | 6.13 |  |  |  |

***Table E13-E14. Parameters obtained by linear regression models*** *between end-inspiratory intrathoracic pressure and* ***corrected*** ***end-inspiratory esophageal pressure*** *according to the catheter tested (esophageal balloon inflated at V_BEST_). Regressions were performed for the overall data, for different catheters with overall PEEP values applied and at each individual PEEP value (0, 5, 10 and 15 cmH_2_O). Equation: f = y0+a*x. Parm. indicates parameters, CI indicates confidence interval, Rsqr indicates R*^2^*, Adj indicates adjusted.*

| **catheter** | **procedure** | **PEEP** | **Coefficient** | **Parm** | **Std. Error** | **t** | ***P*** | **95% CI** | | **Rsqr** | **Adj Rsqr** | ***P*** |
| --- | --- | --- | --- | --- | --- | --- | --- | --- | --- | --- | --- | --- |
| **ALL** | **ALL** | **ALL** | **y0** | -0.33 | 0.80 | -0.42 | 0.677 | -1.92 | 1.25 | 0.85 | 0.85 | <0.001 |
|  |  |  | **a** | 1.12 | 0.04 | 25.58 | <0.001 | 1.03 | 1.20 |  |  |  |
| **COOPER** | **MANUAL INCREMENTAL STEP INFLATION** | **ALL** | **y0** | 0.16 | 2.07 | 0.08 | 0.937 | -4.18 | 4.51 | 0.85 | 0.84 | <0.001 |
|  |  |  | **a** | 1.16 | 0.12 | 9.92 | <0.001 | 0.92 | 1.41 |  |  |  |
|  |  | **0** | **y0** | -1.22 | 1.00 | -1.23 | 0.308 | -4.40 | 1.95 | 0.96 | 0.95 | 0.003 |
|  |  |  | **a** | 1.21 | 0.14 | 8.90 | 0.003 | 0.78 | 1.64 |  |  |  |
|  |  | **5** | **y0** | -0.28 | 1.59 | -0.17 | 0.873 | -5.35 | 4.79 | 0.95 | 0.93 | 0.005 |
|  |  |  | **a** | 1.12 | 0.15 | 7.63 | 0.005 | 0.65 | 1.59 |  |  |  |
|  |  | **10** | **y0** | 3.79 | 4.82 | 0.79 | 0.489 | -11.56 | 19.14 | 0.80 | 0.73 | 0.041 |
|  |  |  | **a** | 0.99 | 0.29 | 3.46 | 0.041 | 0.08 | 1.91 |  |  |  |
|  |  | **15** | **y0** | 55.62 | 14.67 | 3.79 | 0.032 | 8.93 | 102.31 | 0.43 | 0.24 | 0.229 |
|  |  |  | **a** | -0.79 | 0.52 | -1.50 | 0.229 | -2.45 | 0.88 |  |  |  |

| **catheter** | **procedure** | **PEEP** | **Coefficient** | **Parm** | **Std. Error** | **t** | ***P*** | **95% CI** | | **Rsqr** | **Adj Rsqr** | ***P*** |
| --- | --- | --- | --- | --- | --- | --- | --- | --- | --- | --- | --- | --- |
| **ALL** | **ALL** | **ALL** | **y0** | -0.33 | 0.80 | -0.42 | 0.677 | -1.92 | 1.25 | 0.85 | 0.85 | <0.001 |
|  |  |  | **a** | 1.12 | 0.04 | 25.58 | <0.001 | 1.03 | 1.20 |  |  |  |
| **NUTRIVENT** | **MANUAL INCREMENTAL STEP INFLATION** | **ALL** | **y0** | -1.74 | 1.47 | -1.18 | 0.252 | -4.82 | 1.35 | 0.92 | 0.92 | <0.001 |
|  |  |  | **a** | 1.16 | 0.08 | 14.84 | <0.001 | 1.00 | 1.33 |  |  |  |
|  |  | **0** | **y0** | 1.65 | 0.89 | 1.84 | 0.162 | -1.20 | 4.50 | 0.94 | 0.93 | 0.006 |
|  |  |  | **a** | 0.76 | 0.11 | 7.13 | 0.006 | 0.42 | 1.10 |  |  |  |
|  |  | **5** | **y0** | 2.55 | 1.09 | 2.33 | 0.102 | -0.93 | 6.03 | 0.96 | 0.95 | 0.003 |
|  |  |  | **a** | 0.77 | 0.09 | 8.47 | 0.003 | 0.48 | 1.05 |  |  |  |
|  |  | **10** | **y0** | 8.19 | 6.94 | 1.18 | 0.323 | -13.89 | 30.27 | 0.45 | 0.27 | 0.214 |
|  |  |  | **a** | 0.59 | 0.37 | 1.57 | 0.214 | -0.60 | 1.78 |  |  |  |
|  |  | **15** | **y0** | 33.06 | 37.93 | 0.87 | 0.448 | -87.65 | 153.78 | 0.00 | -0.33 | 0.996 |
|  |  |  | **a** | -0.01 | 1.31 | -0.01 | 0.996 | -4.18 | 4.16 |  |  |  |

***Table E15-E16. Parameters obtained by linear regression models*** *between intrathoracic pressure swings and* ***esophageal pressure swings*** *according to the catheter tested. Regressions were performed for the overall data, for different catheters with overall PEEP values applied and at each individual PEEP value (0, 5, 10 and 15 cmH_2_O). Equation: f = y0+a*x. Parm. indicates parameters, CI indicates confidence interval, Rsqr indicates R*^2^*, Adj indicates adjusted.*

| **catheter** | **procedure** | **PEEP** | **Coefficient** | **Parm** | **Std. Error** | **t** | ***P*** | **95% CI** | | **Rsqr** | **Adj Rsqr** | ***P*** |
| --- | --- | --- | --- | --- | --- | --- | --- | --- | --- | --- | --- | --- |
| **ALL** | **ALL** | **ALL** | **y0** | -0.77 | 0.56 | -1.37 | 0.172 | -1.89 | 0.34 | 0.74 | 0.74 | <0.001 |
|  |  |  | **a** | 1.13 | 0.06 | 18.39 | <0.001 | 1.01 | 1.25 |  |  |  |
| **COOPER** | **MANUAL INCREMENTAL STEP INFLATION** | **ALL** | **y0** | -0.93 | 1.33 | -0.70 | 0.491 | -3.73 | 1.86 | 0.78 | 0.77 | <0.001 |
|  |  |  | **a** | 1.33 | 0.16 | 8.05 | <0.001 | 0.98 | 1.67 |  |  |  |
|  |  | **0** | **y0** | 3.80 | 2.30 | 1.65 | 0.197 | -3.53 | 11.13 | 0.03 | -0.29 | 0.764 |
|  |  |  | **a** | 0.14 | 0.44 | 0.33 | 0.764 | -1.25 | 1.54 |  |  |  |
|  |  | **5** | **y0** | -1.11 | 1.23 | -0.90 | 0.432 | -5.03 | 2.81 | 0.88 | 0.84 | 0.018 |
|  |  |  | **a** | 1.32 | 0.28 | 4.68 | 0.018 | 0.42 | 2.23 |  |  |  |
|  |  | **10** | **y0** | -0.99 | 2.76 | -0.36 | 0.743 | -9.76 | 7.78 | 0.79 | 0.71 | 0.045 |
|  |  |  | **a** | 1.53 | 0.46 | 3.31 | 0.045 | 0.06 | 2.99 |  |  |  |
|  |  | **15** | **y0** | 18.03 | 17.09 | 1.05 | 0.369 | -36.37 | 72.42 | 0.00 | -0.33 | 0.961 |
|  |  |  | **a** | -0.07 | 1.28 | -0.05 | 0.961 | -4.14 | 4.00 |  |  |  |

| **catheter** | **procedure** | **PEEP** | **Coefficient** | **Parm** | **Std. Error** | **t** | ***P*** | **95% CI** | | **Rsqr** | **Adj Rsqr** | ***P*** |
| --- | --- | --- | --- | --- | --- | --- | --- | --- | --- | --- | --- | --- |
| **ALL** | **ALL** | **ALL** | **y0** | -0.77 | 0.56 | -1.37 | 0.172 | -1.89 | 0.34 | 0.74 | 0.74 | <0.001 |
|  |  |  | **a** | 1.13 | 0.06 | 18.39 | <0.001 | 1.01 | 1.25 |  |  |  |
| **NUTRIVENT** | **MANUAL INCREMENTAL STEP INFLATION** | **ALL** | **y0** | -2.37 | 1.15 | -2.06 | 0.054 | -4.78 | 0.05 | 0.88 | 0.87 | <0.001 |
|  |  |  | **a** | 1.34 | 0.12 | 11.25 | <0.001 | 1.09 | 1.59 |  |  |  |
|  |  | **0** | **y0** | -1.35 | 0.39 | -3.48 | 0.040 | -2.58 | -0.11 | 0.99 | 0.99 | <0.001 |
|  |  |  | **a** | 1.10 | 0.05 | 20.53 | <0.001 | 0.93 | 1.27 |  |  |  |
|  |  | **5** | **y0** | -0.80 | 0.46 | -1.74 | 0.180 | -2.26 | 0.66 | 0.99 | 0.98 | 0.00 |
|  |  |  | **a** | 1.06 | 0.07 | 15.00 | 0.001 | 0.84 | 1.29 |  |  |  |
|  |  | **10** | **y0** | 3.23 | 3.03 | 1.07 | 0.365 | -6.41 | 12.87 | 0.56 | 0.41 | 0.15 |
|  |  |  | **a** | 0.70 | 0.36 | 1.95 | 0.146 | -0.44 | 1.84 |  |  |  |
|  |  | **15** | **y0** | 1.01 | 22.11 | 0.05 | 0.967 | -69.35 | 71.36 | 0.15 | -0.13 | 0.51 |
|  |  |  | **a** | 1.14 | 1.54 | 0.74 | 0.512 | -3.76 | 6.04 |  |  |  |

### Table E17-E19. Estimated parameters of each linear regression model and comparison with those of identity line (intercept=0 and regression coefficient =1) (F test), according to pressures.

***Table E17:*** *Relationship between end-expiratory intrathoracic pressures and end-expiratory esophageal pressures (uncorrected and corrected). Esophageal balloon inflated at V_BEST_. Rsqr indicates R^2^, Int indicates intercept.*

| **Correction** | **Catheter** | **Procedure** | **Cefficient** | | **Rsqr** | ***P*** | ***P***  **(slope=1)** | ***P***  **(Int=0)** |
| --- | --- | --- | --- | --- | --- | --- | --- | --- |
| **NO** | **COOPER** | **MANUAL INCREMENTAL STEP INFLATION** | **Intercept** | 3.11 | 0.88 | <0.001 | 0.068 | 0.008 |
|  |  |  | **Slope** | 1.21 |  |  |  |  |
| **YES** | **COOPER** | **MANUAL INCREMENTAL STEP INFLATION** | **Intercept** | 1.26 | 0.90 | <0.001 | 0.926 | 0.129 |
|  |  |  | **Slope** | 1.01 |  |  |  |  |
| **NO** | **NUTRIVENT** | **MANUAL INCREMENTAL STEP INFLATION** | **Intercept** | 2.09 | 0.84 | <0.001 | 0.014 | 0.147 |
|  |  |  | **Slope** | 1.39 |  |  |  |  |
| **YES** | **NUTRIVENT** | **MANUAL INCREMENTAL STEP INFLATION** | **Intercept** | 0.30 | 0.95 | <0.001 | 0.973 | 0.567 |
|  |  |  | **Slope** | 1.00 |  |  |  |  |

***Table E18:*** *Relationship between end-inspiratory intrathoracic pressures and end-inspiratory esophageal pressures (uncorrected and corrected) Esophageal balloon inflated at V_BEST_. Rsqr indicates R^2^, Int indicates intercept.*

| **Correction** | **Catheter** | **Procedure** | **Cefficient** | | **Rsqr** | ***P*** | ***P* (Slope=1)** | ***P***  **(Int=0)** |
| --- | --- | --- | --- | --- | --- | --- | --- | --- |
| **NO** | **COOPER** | **MANUAL INCREMENTAL STEP INFLATION** | **Intercept** | 1.90 | 0.84 | <0.001 | 0.049 | 0.428 |
|  |  |  | **Slope** | 1.28 |  |  |  |  |
| **YES** | **COOPER** | **MANUAL INCREMENTAL STEP INFLATION** | **Intercept** | 0.16 | 0.85 | <0.001 | 0.178 | 0.937 |
|  |  |  | **Slope** | 1.16 |  |  |  |  |
| **NO** | **NUTRIVENT** | **MANUAL INCREMENTAL STEP INFLATION** | **Intercept** | -1.08 | 0.86 | <0.001 | 0.007 | 0.672 |
|  |  |  | **Slope** | 1.41 |  |  |  |  |
| **YES** | **NUTRIVENT** | **MANUAL INCREMENTAL STEP INFLATION** | **Intercept** | -1.74 | 0.92 | <0.001 | 0.052 | 0.252 |
|  |  |  | **Slope** | 1.16 |  |  |  |  |

***Table E19:*** *Relationship between intrathoracic pressure swings and esophageal pressure swings. Rsqr indicates R^2^, Int indicates intercept; NA indicates not applicable.*

| **Correction** | **Catheter** | **Procedure** | **Cefficient** | | **Rsqr** | ***P*** | ***P* (Slope=1)** | ***P***  **(Int=0)** |
| --- | --- | --- | --- | --- | --- | --- | --- | --- |
| **NA** | **COOPER** | **MANUAL INCREMENTAL STEP INFLATION** | **Intercept** | -0.93 | 0.78 | <0.001 | 0.064 | 0.492 |
|  |  |  | **Slope** | 1.33 |  |  |  |  |
| **NA** | **NUTRIVENT** | **MANUAL INCREMENTAL STEP INFLATION** | **Intercept** | -2.37 | 0.88 | <0.001 | 0.010 | 0.054 |
|  |  |  | **Slope** | 1.34 |  |  |  |  |

### MODEL SELECTION

**Table E20. RESULTS OF THE FIXED EFFECTS SELECTION INTO THE GENERAL LINEAR MIXED MODELS ON ABSOLUTE VALUES.**

*The dependent variables were the difference between esophageal pressure and intrathoracic pressures at end-expiration and end-inspiration. The model was implemented including, first, only* ***CATHETER (Cooper, Nutrivent), PEEP (0, 5, 10, 15 cmH_2_O) and CORRECTION (YES, NO)*** *and then adding the interactions between couples of effects. The best model (yellow cells) was chosen according to AIC and to the parsimony principle for mathematical models. Esophageal balloon inflated at V_BEST_*

| **PROCEDURE** |  |  | **FIXED EFFECTS** | | | | | | | |
| --- | --- | --- | --- | --- | --- | --- | --- | --- | --- | --- |
|  |  |  | **catheter PEEP correction** | **catheter PEEP correction catheter*PEEP** | **catheter PEEP correction catheter*correction** | **catheter PEEP correction PEEP*correction** | **catheter PEEP correction PEEP*correction catheter*correction** | **catheter PEEP correction PEEP*correction catheter*PEEP** | **catheter PEEP correction**  **catheter*correction catheter*PEEP** | **catheter PEEP correction catheter*correction catheter*PEEP**  **PEEP*correction** |
| **MANUAL INCREMENTAL STEP INFLATION** | **P_EE_** | **AIC** | 348.44 | 341.56 | 344.37 | **324.82** | 320.07 | 318.56 | 337.54 | 313.89 |
|  |  | **Statistically significant** | correction | correction | PEEP correction | **PEEP correction PEEP***  **correction** | PEEP correction PEEP*  correction | PEEP correction PEEP*  correction | PEEP correction | PEEP correction PEEP*  correction |
|  | **P_EI_** | **AIC** | 401.53 | 392.97 | 397.94 | **382.45** | 378.73 | 374.19 | 389.4 | 370.49 |
|  |  | **Statistically significant** | PEEP correction | PEEP correction | PEEP correction | **PEEP correction PEEP***  **correction** | PEEP correction PEEP*  correction | PEEP correction PEEP*  correction | PEEP correction | PEEP correction PEEP*  correction |

**Table E21. RESULTS OF THE FIXED EFFECTS SELECTION INTO THE GENERAL LINEAR MIXED MODELS ON SWINGS VALUES.**

*The dependent variables were the difference between esophageal pressure swings and intrathoracic pressure swings. The model was implemented including, first, only* ***CATHETER (Cooper, Nutrivent) AND PEEP (0, 5, 10, 15 cmH_2_O)*** *and then including the interaction between the effects. The best model (yellow cells) was chosen according to AIC and to the parsimony principle for mathematical models.*

| **PROCEDURE** |  |  | **FIXED EFFECTS** | |
| --- | --- | --- | --- | --- |
|  |  |  | **catheter PEEP** | **catheter PEEP catheter*PEEP** |
| **MANUAL INCREMENTAL STEP INFLATION** | **PRESSURE SWINGS** | **AIC** | **160.11** | 152.81 |
|  |  | **Statistically significant** | **PEEP** | PEEP |

### RESULTS OF GENERAL LINEAR MIXED MODELS APPLIED TO END-EXPIRATORY PRESSURE DURING MANUAL INCREMENTAL STEP INFLATION

*Esophageal balloon inflated at V_BEST_*

Figure E16 (Figure 3 in main paper) shows the relationship (overall and according to PEEP) between end-expiratory esophageal pressure obtained by manual incremental step inflation and intrathoracic pressure (panels A, B, C, D) and their difference (panels E and F) according to the catheters tested (Cooper, panels A, C, E; Nutrivent panels B, D, F). Panels A and B refer to uncorrected end-expiratory esophageal pressure while panels C and D represent corrected values. Both uncorrected (Cooper: R^2^=0.88; Nutrivent: R^2^=0.84) and corrected (Cooper: R^2^=0.90; Nutrivent: R^2^=0.95) end-expiratory esophageal pressure values are related to intrathoracic pressure values but corrected values better approximate the identity line (see table E17).

The difference computed between end-expiratory esophageal pressure measured by manual incremental step inflation and intrathoracic pressure was not statistically different between the two catheters tested (*P*= 0.4608). The correction application on end-expiratory esophageal pressure significantly reduced the overall difference with intrathoracic pressure values from 5.01±3.32 to 0.82±1.62 cmH_2_O (*P*= 0.0003). PEEP, independently on the other factors tested, affected the measured difference (1.48±1.8, 2.11±1.99, 3.49±3.46, 4.58±4.60 cmH_2_O, at PEEP 0, 5, 10 and 15 cmH_2_O respectively, *P*= 0.0392) and there was a significant interaction between PEEP and correction application (*P*=0.0002). At each PEEP value applied, differences computed with uncorrected esophageal pressure are always higher than values computed after correction application and, independently on the catheter used, increased with PEEP (2.36±2.03, 3.77±1.37, 6.24±2.51, 7.69±4.02 cmH_2_O, at PEEP 0, 5, 10 and 15 cmH_2_O respectively, *P* <.0001). On the contrary differences computed after correction application were not correlated with PEEP values and were not statistically different from 0 (0.60±1.09, 0.45±0.60, 0.74±1.48, 1.47±2.64 cmH_2_O, at PEEP 0, 5, 10 and 15 cmH_2_O respectively, *P*= 0.8115).

**Figure E16.**

*Figure* *shows the relationship (overall and according to different PEEP levels) between end-expiratory esophageal pressure obtained by manual incremental step inflation and end-expiratory intrathoracic pressure (panels* ***A, B, C, D****) and their difference (panels* ***E*** *and* ***F****) according to the tested catheters (Cooper, panels* ***A, C, E****; Nutrivent panels* ***B, D, F****). Panels* ***A*** *and* ***B*** *refer to uncorrected end-expiratory esophageal pressure while panels* ***C*** *and* ***D*** *refer to corrected values. Blue colour represents PEEP 0 cmH_2_O; dark-red colour represents PEEP 5 cmH_2_O; green colour represents PEEP 10 cmH_2_O; dark-yellow colour represents PEEP 15 cmH_2_O. Blue, dark-red, green and dark-yellow lines represent linear regressions at different PEEP values, dark-grey line represents linear regression at all PEEP levels. Black continuous line represents the identity line. * P<0.05 vs "corrected"; *** P<0.001 vs "corrected"; †† P<0.01 vs PEEP 0; ††† P<0.001 vs PEEP 0; ‡‡ P<0.01 vs PEEP 5.*

### RESULTS OF GENERAL LINEAR MIXED MODELS APPLIED TO END-INSPIRATORY PRESSURE DURING MANUAL INCREMENTAL STEP INFLATION

*Esophageal balloon inflated at V_BEST_*

Figure E17 (Figure 4 in main paper) shows the relationship (overall and according to PEEP) between end-inspiratory esophageal pressure obtained by manual incremental step inflation and intrathoracic pressure (panels A, B, C, D) and their difference (panels E and F) according to the catheters tested (Cooper, panels A, C, E; Nutrivent panels B, D, F). Panels A and B refer to uncorrected end inspiratory esophageal pressure while panels C and D represent corrected values. Both uncorrected (Cooper: R^2^=0.84; Nutrivent: R^2^=0.86) and corrected (Cooper: R^2^=0.85; Nutrivent: R^2^=0.92) end-inspiratory esophageal pressure values are related to intrathoracic pressure values, corrected values better approximate the identity line. (see table E18).

The difference computed between end-inspiratory esophageal pressure measured by manual incremental step inflation and intrathoracic pressure was not statistically different between the two catheters tested (*P*=0.2069). The correction application on end-expiratory esophageal pressure significantly reduced the overall difference with intrathoracic pressure values from 6.06±5.62 to 1.86±3.94 cmH_2_O (*P*=0.0010). Higher PEEP, independently on the other factors tested, increased the measured difference (0.84±1.72, 2.04±2.19, 4.92±4.18, 8.03±7.57 cmH_2_O, at PEEP 0, 5, 10 and 15 cmH_2_O respectively, *P*=0.0219) and, similarly to end-expiratory values, there was a significant interaction with correction application (*P*=0.0084). Differences computed with uncorrected esophageal pressure were higher than values computed after correction application at 5 (*P*=0.0014), 10 (*P*<0.001) and 15 (*P*<0.001) cmH_2_O PEEP and, independently on the catheter used, increased with PEEP (1.71±2.01, 3.70±1.73, 7.67±3.62, 11.14±7.60 cmH_2_O, at PEEP 0, 5, 10 and 15 cmH_2_O respectively, *P*=0.0004). On the contrary differences computed after correction application were not significantly associated with PEEP values (-0.04±0.69, 0.38±1.00, 2.17±2.63, 4.92±6.47, at PEEP 0, 5, 10 and 15 respectively, *P*=0.1155).

**Figure E17.**

*Figure* *shows the relationship (overall and according to different PEEP levels) between end-inspiratory esophageal pressure obtained by manual incremental step inflation and end-inspiratory intrathoracic pressure (panels* ***A, B, C, D****) and their difference (panels* ***E*** *and* ***F****) according to the tested catheters (Cooper, panels* ***A, C, E****; Nutrivent panels* ***B, D, F****). Panels* ***A*** *and* ***B*** *refer to uncorrected end- inspiratory esophageal pressure while panels* ***c*** *and* ***d*** *refer to corrected values. Blue colour represents PEEP 0 cmH_2_O; dark-red colour represents PEEP 5 cmH_2_O; green colour represents PEEP 10 cmH_2_O; dark-yellow colour represents PEEP 15 cmH_2_O. Blue, dark-red, green and dark-yellow lines represent linear regressions at different PEEP values, dark-grey line represents linear regression at all PEEP levels. Black continuous line represents the identity line. ** P<0.01 vs "corrected"; *** P<0.001 vs "corrected"; ††† P<0.001 vs PEEP 0; ‡‡ P<0.01 vs PEEP 5.*

### RESULTS OF GENERAL LINEAR MIXED MODELS APPLIED TO PRESSURE SWINGS DURING MANUAL INCREMENTAL STEP INFLATION

*Esophageal balloon inflated at V_BEST_*

Figure E18 (figure 5 in the main manuscript) shows the relationship (overall and according to PEEP) between esophageal pressure swings obtained by manual incremental step inflation and intrathoracic pressure swings (panels A, B) and their difference (panels C and D) according to the catheters tested (Cooper, panels A, C; Nutrivent panels B, D). Esophageal pressure swings values are related to intrathoracic pressure swings values (Cooper: R^2^=0.78; Nutrivent: R^2^=0.88) but the slope values were 1.33 for Cooper and 1.34 for Nutrivent due to pressures at higher PEEP (see table E19).

The difference computed between esophageal pressure swings measured by manual incremental step inflation and intrathoracic pressure swings was not statistically different between the two catheters tested (*P*=0.1651). The measured difference increased with PEEP (-0.64±0.62, -0.07±0.53, 1.43±1.51, 3.45±3.94 cmH_2_O, at PEEP 0, 5, 10 and 15 cmH_2_O respectively, *P*=0.0197).

**Figure E18.**

*Figure* *shows the relationship (overall and according to different PEEP levels) between esophageal pressure swings obtained by manual incremental step inflation and intrathoracic pressure swings (panels* ***A, B****) and their difference (panels* ***C*** *and* ***D****) according to the tested catheters (Cooper, panels* ***A, C****; Nutrivent panels* ***B, D****). Blue colour represents PEEP 0 cmH_2_O; dark-red colour represents PEEP 5 cmH_2_O; green colour represents PEEP 10 cmH_2_O; dark-yellow colour represents PEEP 15 cmH_2_O. Blue, dark-red, green and dark-yellow lines represent linear regressions at different PEEP values, dark-grey line represents linear regression at all PEEP levels. Black continuous line represents the identity line. † P<0.05 vs PEEP 0.*

### COMPARISON BETWEEN ESOPHAGUS-ESOPHAGEAL BALLOON ELASTANCES AT THE BEGINNING AND AT THE END OF THE STUDY

*Esophageal balloon inflated at V_BEST_*

**Figure E19:** *Figure represents the esophagus+esophageal balloon volume-pressure curves at 0 cmH_2_O PEEP measured with Cooper catheter for pig n°6 (panel A) and n°8 (panel C) and Nutrivent catheter for pig n°7 (Panel B). Blue lines represent the beginning of the experiments (START), red line the end (END). Black lines represent the sigmoidal fitting of the experimental data and yellow dots represent the limits of the quasi-linear part of the fitting curves. Lower limits of the quasi-linear portion of the curve for pig n° 7 were out the experimental data range, therefore the minimum experimental values of pressures and volumes were used.*

### PRODUCER VOLUME

### MODEL SELECTION

**Table E23. RESULTS OF THE FIXED EFFECTS SELECTION INTO THE GENERAL LINEAR MIXED MODELS ON ABSOLUTE VALUES.**

*The dependent variables were the difference between esophageal pressure and intrathoracic pressures at end-expiration and end-inspiration. The model was implemented including, first, only* ***CATHETER (Cooper, Nutrivent), PEEP (0, 5, 10, 15 cmH_2_O) and CORRECTION (YES, NO)*** *and then adding the interactions between couples of effects. The best model (yellow cells) was chosen according to AIC and to the parsimony principle for mathematical models.*

| **PROCEDURE** |  |  | **FIXED EFFECTS** | | | | | | | |
| --- | --- | --- | --- | --- | --- | --- | --- | --- | --- | --- |
|  |  |  | **catheter PEEP correction** | **catheter PEEP correction catheter*PEEP** | **catheter PEEP correction catheter*correction** | **catheter PEEP correction PEEP*correction** | **catheter PEEP correction PEEP*correction catheter*correction** | **catheter PEEP correction PEEP*correction catheter*PEEP** | **catheter PEEP correction**  **catheter*correction catheter*PEEP** | **catheter PEEP correction catheter*correction catheter*PEEP**  **PEEP*correction** |
| **MANUAL INCREMENTAL STEP INFLATION** | **P_EE_** | **AIC** | 292.22 | 288.00 | **271.17** | 287.15 | 266.29 | 280.89 | 267.59 | 262.72 |
|  |  | **Statistically significant** | correction | correction | **correction**  **catheter***  **correction** | correction | correction catheter*  correction | correction | correction  catheter*  correction | correction catheter*  correction |
|  | **P_EI_** | **AIC** | 350.94 | 342.03 | **340.11** | 344.87 | 334.35 | 336.00 | 330.98 | 325.27 |
|  |  | **Statistically significant** | correction | correction | **correction**  **catheter***  **correction** | correction | correction catheter*  correction | correction | correction  catheter*  correction | correction catheter*  correction |

**Table E24. RESULTS OF THE FIXED EFFECTS SELECTION INTO THE GENERAL LINEAR MIXED MODELS ON SWINGS VALUES.**

*The dependent variables were the difference between esophageal pressure swings and intrathoracic pressure swings. The model was implemented including, first, only* ***CATHETER (Cooper, Nutrivent)*** *and* ***PEEP (0, 5, 10, 15 cmH_2_O)*** *and then including the interaction between them effects. The best model (yellow cells) was chosen according to AIC and to the parsimony principle for mathematical models.*

| **PROCEDURE** |  |  | **FIXED EFFECTS** | |
| --- | --- | --- | --- | --- |
|  |  |  | **catheter PEEP** | **catheter PEEP catheter*PEEP** |
| **MANUAL INCREMENTAL STEP INFLATION** | **PRESSURE SWINGS** | **AIC** | **144.57** | 138.04 |
|  |  | **Statistically significant** | **none** | none |

### RESULTS OF GENERAL LINEAR MIXED MODELS APPLIED TO END-EXPIRATORY PRESSURE DURING MANUAL INCREMENTAL STEP INFLATION - Esophageal balloon inflated at v_producer_

Figure E20 shows the relationship (overall and according to PEEP) between end-expiratory esophageal pressure obtained by manual incremental step inflation and intrathoracic pressure (panels A, B, C, D) and their difference (panels E and F) according to the catheters tested (Cooper, panels A, C, E; Nutrivent panels B, D, F). Panels A and B refer to uncorrected end-expiratory esophageal pressure while panels C and D represent corrected values. Both uncorrected and corrected end-expiratory esophageal pressure values are associated to intrathoracic pressure values. Corrected values better approximate the identity lines.

| **Correction** | **Catheter** | **Procedure** | **Coefficient** | | **Rsqr** | ***P*** | ***P***  **(slope=1)** | ***P***  **(Int=0)** |
| --- | --- | --- | --- | --- | --- | --- | --- | --- |
| **NO** | **COOPER** | **MANUAL INCREMENTAL STEP INFLATION** | **Intercept** | 2.54 | 0.89 | <.0001 | 0.911 | 0.006 |
|  |  |  | **Slope** | 1.01 |  |  |  |  |
| **YES** | **COOPER** | **MANUAL INCREMENTAL STEP INFLATION** | **Intercept** | 1.21 | 0.89 | <.0001 | 0.835 | 0.157 |
|  |  |  | **Slope** | 1.02 |  |  |  |  |
| **NO** | **NUTRIVENT** | **MANUAL INCREMENTAL STEP INFLATION** | **Intercept** | 2.66 | 0.90 | <.0001 | 0.280 | 0.004 |
|  |  |  | **Slope** | 1.09 |  |  |  |  |
| **YES** | **NUTRIVENT** | **MANUAL INCREMENTAL STEP INFLATION** | **Intercept** | -5.88e-3 | 0.95 | <.0001 | 0.972 | 0.991 |
|  |  |  | **Slope** | 1.00 |  |  |  |  |

***Table E25****: Estimated parameters of each linear regression model between end-expiratory intrathoracic pressures and end-expiratory esophageal pressures (uncorrected and corrected) and comparison with those of identity line (intercept=0 and regression coefficient =1) (F test), according to pressures. Rsqr indicates R^2^, Int indicates intercept.*

The difference computed between end-expiratory esophageal pressure measured by manual incremental step inflation and intrathoracic pressure was not statistically different between the two catheters tested (*P*=0.3369). PEEP, independently on the other factors tested, did not affect the measured difference (*P*= 0.3019). The correction application on end-expiratory esophageal pressure significantly reduced the overall difference with intrathoracic pressure values (uncorrected value, 3.01±1.95; corrected value, 0.67±1.74 mH_2_O, *P*=0.0016). There was a significant interaction between the catheter tested and correction application (*P*<.0001). Correction effect is significantly different testing catheters separately (Cooper, uncorrected value 2.62±1.87 cmH_2_O; corrected value 1.35±1.89 cmH_2_O. *P*=0.0015; Nutrivent, uncorrected value 3.41±1.99 cmH_2_O; corrected value -0.02±1.28 cmH_2_O. *P*<.0001). Catheters effect in uncorrected and corrected values separately was statistically significant (*P*=0.0258 for uncorrected values, *P*=0.0002 for corrected values).

**FIGURE E20**

*Figure* *shows the relationship (overall and according to different PEEP levels) between end-expiratory esophageal pressure obtained by incremental step inflation and end-expiratory intrathoracic pressure (panels* ***A, B, C, D****) and their difference (panels* ***E*** *and* ***F****) according to the tested catheters (Cooper, panels* ***A, C, E****; Nutrivent panels* ***B, D, F****). Panels* ***A*** *and* ***B*** *refer to uncorrected end-expiratory esophageal pressure while panels* ***C*** *and* ***D*** *refer to corrected values. Blue colour represents PEEP 0 cmH_2_O; dark-red colour represents PEEP 5 cmH_2_O; green colour represents PEEP 10 cmH_2_O; dark-yellow colour represents PEEP 15 cmH_2_O. Blue, dark-red, green and dark-yellow lines represent linear regressions at different PEEP values, dark-grey line represents linear regression at all PEEP levels. Black continuous line represents the identity line.*

### RESULTS OF GENERAL LINEAR MIXED MODELS APPLIED TO END-INSPIRATORY PRESSURE DURING MANUAL INCREMENTAL STEP INFLATION - Esophageal balloon inflated at v_producer_

Figure E21 shows the relationship (overall and according to PEEP) between end-inspiratory esophageal pressure obtained by manual incremental step inflation and intrathoracic pressure (panels A, B, C, D) and their difference (panels E and F) according to the catheters tested (Cooper, panels A, C, E; Nutrivent panels B, D, F). Panels A and B refer to uncorrected end-inspiratory esophageal pressure while panels C and D represent corrected values. Both uncorrected and corrected end-inspiratory esophageal pressure values are associated to intrathoracic pressure values.

| **Correction** | **Catheter** | **Procedure** | **Cefficient** | | **Rsqr** | ***P*** | ***P***  **(slope=1)** | ***P***  **(Int=0)** |
| --- | --- | --- | --- | --- | --- | --- | --- | --- |
| **NO** | **COOPER** | **MANUAL INCREMENTAL STEP INFLATION** | **Intercept** | 1.77 | 0.86 | <.0001 | 0.377 | 0.354 |
|  |  |  | **Slope** | 1.10 |  |  |  |  |
| **YES** | **COOPER** | **MANUAL INCREMENTAL STEP INFLATION** | **Intercept** | 0.49 | 0.86 | <.0001 | 0.370 | 0.793 |
|  |  |  | **Slope** | 1.1 |  |  |  |  |
| **NO** | **NUTRIVENT** | **MANUAL INCREMENTAL STEP INFLATION** | **Intercept** | 0.87 | 0.91 | <.0001 | 0.085 | 0.606 |
|  |  |  | **Slope** | 1.16 |  |  |  |  |
| **YES** | **NUTRIVENT** | **MANUAL INCREMENTAL STEP INFLATION** | **Intercept** | -1.61 | 0.94 | <.0001 | 0.133 | 0.215 |
|  |  |  | **Slope** | 1.1 |  |  |  |  |

***Table E26:*** *Estimated parameters of each linear regression model between end-inspiratory intrathoracic pressures and end-inspiratory esophageal pressures (uncorrected and corrected) and comparison with those of identity line (intercept=0 and regression coefficient =1) (F test), according to pressures. Rsqr indicates R^2^, Int indicates intercept.*

The difference computed between end-inspiratory esophageal pressure measured by manual incremental step inflation and intrathoracic pressure was not statistically different between the two catheters tested (*P*=0.1771). PEEP, independently on the other factors tested, did not affect the measured difference (*P*=0.1594). The correction application on end-inspiratory esophageal pressure significantly reduced the overall difference with intrathoracic pressure values (uncorrected value, 4.42± 3.66 cmH_2_O; corrected value 1.07± 3.38 cmH_2_O, *P*=0.0021). There was a significant interaction between the catheter tested and correction application (*P*=0.0021). Correction effect is significant testing catheters separately (Cooper, uncorrected value 3.24±3.94 cmH_2_O; corrected value 1.98±3.92 cmH_2_O. *P*=0.0100; Nutrivent, uncorrected value 3.59±3.44 cmH_2_O; corrected value 0.16±2.54 cmH_2_O. *P*<.0001). Catheters effect in uncorrected values was not statistically significant (*P*=0.5336) while it was significant in corrected values *P*=0.0021).

**FIGURE E21**

*Figure* *shows the relationship (overall and according to different PEEP levels) between end-inspiratory esophageal pressure obtained by incremental step inflation and end-inspiratory intrathoracic pressure (panels* ***A, B, C, D****) and their difference (panels* ***E*** *and* ***F****) according to the tested catheters (Cooper, panels* ***A, C, E****; Nutrivent panels* ***B, D, F****). Panels* ***A*** *and* ***B*** *refer to uncorrected end-inspiratory esophageal pressure while panels* ***C*** *and* ***D*** *refer to corrected values. Blue colour represents PEEP 0 cmH_2_O; dark-red colour represents PEEP 5 cmH_2_O; green colour represents PEEP 10 cmH_2_O; dark-yellow colour represents PEEP 15 cmH_2_O. Blue, dark-red, green and dark-yellow lines represent linear regressions at different PEEP values, dark-grey line represents linear regression at all PEEP levels. Black continuous line represents the identity line.*

### RESULTS OF GENERAL LINEAR MIXED MODELS APPLIED TO PRESSURE SWINGS DURING MANUAL INCREMENTAL STEP INFLATION - Esophageal balloon inflated at v_producer_

Figure E22 shows the relationship (overall and according to PEEP) between esophageal pressure swings obtained by manual incremental step inflation and intrathoracic pressure swings (panels A, B) and their difference (panels C and D) according to the catheters tested (Cooper, panels A, C; Nutrivent panels B, D). Esophageal pressure swings values are associated to intrathoracic pressure swings values but slopes are higher than 1.

| **Correction** | **Catheter** | **Procedure** | **Cefficient** | | **Rsqr** | ***P*** | ***P* (Slope=1)** | ***P***  **(Int=0)** |
| --- | --- | --- | --- | --- | --- | --- | --- | --- |
| **NA** | **COOPER** | **MANUAL INCREMENTAL STEP INFLATION** | **Intercept** | -0.48 | 0.81 | <.0001 | <.0001 | 0.039 |
|  |  |  | **Slope** | 1.15 |  |  |  |  |
| **NA** | **NUTRIVENT** | **MANUAL INCREMENTAL STEP INFLATION** | **Intercept** | -1.78 | 0.91 | <.0001 | <.0001 | 0.084 |
|  |  |  | **Slope** | 1.22 |  |  |  |  |

***Table E27****: Estimated parameters of each linear regression model between intrathoracic pressure swings and esophageal pressure swings and comparison with those of identity line (intercept=0 and regression coefficient =1) (F test), according to pressures. Rsqr indicates R^2^, Int indicates intercept..*

The difference computed between esophageal pressure swings measured by manual incremental step inflation and intrathoracic pressure swings was not statistically different between the two catheters tested (Cooper: 0.62±2.26 cmH_2_O; Nutrivent: 0.18±1.57 cmH_2_O; *P*=0.2496) and did not change with PEEP *P*=0.0868).

**Figure E22.**

*Figure* *shows the relationship (overall and according to different PEEP levels) between esophageal pressure swings obtained by manual incremental step inflation and intrathoracic pressure swings (panels* ***A, B****) and their difference (panels* ***C*** *and* ***D****) according to the tested catheters (Cooper, panels* ***A, C****; Nutrivent panels* ***B, D****). Blue colour represents PEEP 0 cmH_2_O; dark-red colour represents PEEP 5 cmH_2_O; green colour represents PEEP 10 cmH_2_O; dark-yellow colour represents PEEP 15 cmH_2_O. Blue, dark-red, green and dark-yellow lines represent linear regressions at different PEEP values, dark-grey line represents linear regression at all PEEP levels. Black continuous line represents the identity line.*

### 
